# Customization of a *DADA2*-based pipeline for fungal internal transcribed spacer 1 (ITS1) amplicon data sets

**DOI:** 10.1172/jci.insight.151663

**Published:** 2022-01-11

**Authors:** Thierry Rolling, Bing Zhai, John Frame, Tobias M. Hohl, Ying Taur

**Affiliations:** 1 Infectious Disease Service, Department of Medicine, and; 2 Immunology Program, Sloan Kettering Institute, Memorial Sloan Kettering Cancer Center (MSKCC), New York, New York, USA.; 3 Division of Infectious Diseases, First Department of Medicine, University Medical Center Hamburg-Eppendorf, Hamburg, Germany.; 4 Weill Cornell Medical College, New York, New York, USA.

**Keywords:** Infectious disease, Microbiology, Fungal infections

## Abstract

Identification and analysis of fungal communities commonly rely on internal transcribed spacer–based (ITS-based) amplicon sequencing. There is no gold standard used to infer and classify fungal constituents since methodologies have been adapted from analyses of bacterial communities. To achieve high-resolution inference of fungal constituents, we customized a DADA2-based pipeline using a mix of 11 medically relevant fungi. While DADA2 allowed the discrimination of ITS1 sequences differing by single nucleotides, quality filtering, sequencing bias, and database selection were identified as key variables determining the accuracy of sample inference. Due to species-specific differences in sequencing quality, default filtering settings removed most reads that originated from *Aspergillus* species, *Saccharomyces cerevisiae,* and *Candida glabrata*. By fine-tuning the quality filtering process, we achieved an improved representation of the fungal communities. By adapting a wobble nucleotide in the ITS1 forward primer region, we further increased the yield of *S. cerevisiae* and *C*. *glabrata* sequences. Finally, we showed that a BLAST-based algorithm based on the UNITE+INSD or the NCBI NT database achieved a higher reliability in species-level taxonomic annotation compared with the naive Bayesian classifier implemented in DADA2. These steps optimized a robust fungal ITS1 sequencing pipeline that, in most instances, enabled species-level assignment of community members.

## Introduction

Amplicon-based sequencing methods have allowed researchers to dissect the composition of the bacterial microbiota in a broad range of environmental and biological samples and have widened our knowledge of host-microbe interactions in health and disease (1). More recently, microbiota research has expanded beyond the bacterial kingdom to encompass fungi, archaea, and viruses. The recognized target for fungal taxonomic profiling is the internal transcribed spacer (ITS) region of the ribosomal DNA (rDNA) (2). Due to sequencing length limitations, only 1 of the 2 subregions ITS1 or ITS2 is commonly used. We have previously shown the potential of an ITS1-based approach to identify the intestinal origin of *Candida* bloodstream infections (3). Here, we demonstrate that customization of this ITS1-based platform can improve the accuracy of fungal species representation.

Currently, most amplicon-based microbiota profiling methods rely on sequencing using an Illumina platform. Starting with the raw Illumina sequences, a pipeline for amplicon analysis includes multiple steps: demultiplexing (which is optional), primer removal, quality filtering, denoising or operational taxonomic unit (OTU) picking, and taxonomic annotation.

Distinguishing biological variation from sequencing errors is one of the most important features of any amplicon pipeline. Historically, this has been done by grouping sequences that are similar by an arbitrary threshold (commonly 97%) into 1 OTU. OTU-based methods are still used widely in the recent mycobiota literature (4–6). By design, this approach precludes the discrimination of sequence variants with less than 3% dissimilarity. By increasing the similarity threshold, a higher amount of false OTUs (pseudo-OTUs) will be called that are due to sequencing error and not to biological variation. To counter these limitations, algorithms that infer exact sequencing variants (amplicon sequencing variants [ASV]) by accounting for sequencing quality scores have been developed. Of these, DADA2 is most widely used (7, 8). Alternatives to DADA2 include Deblur (9) as well as UNOISE3, the most recent update of the UNOISE algorithm (10).

The development of these pipelines provides the technical requirements to discriminate sequencing variants in ITS data sets with high resolution. However, individual components of bioinformatic pipelines for microbiome data analysis have been developed for and validated with bacterial 16S rDNA sequences. When applying these tools to fungal ITS data sets, additional complexities (specific to ITS) must be taken into account. First, in contrast to the near-uniform length of 16S amplicons across bacterial species, the length of ITS amplicons varies substantially, between 150 and over 500 nucleotides, in different fungal species. The difference in ITS amplicon length leads to a varying degree of overlap between forward and reverse reads (11). While the high variability in ITS sequence and length complicates the bioinformatic processing of fungal amplicon data sets, it also enables a high resolution in differentiating distinct fungal taxa (2). Second, fungal taxonomic annotation is complicated further by a rapidly evolving taxonomy with major reclassifications of medically relevant fungal taxa in the last few years (12). The choice of the database for taxonomic annotation needs to reflect this process. UNITE is a commonly used database for fungal annotation specific to the ITS region (13). Recently, the NCBI has started curating a specific ITS reference sequence database, as well (14). Alternatively, non–ITS-specific databases such as NCBI NT (https://www.ncbi.nlm.nih.gov/nucleotide/) can be used as a reference for taxonomic classification.

Here, we show that DADA2, the most frequently used ASV-construction tool, effectively discriminated ITS1 amplicons within a mock community of fungal species that were commonly identified in the human intestinal mycobiota. We further optimized the output of a DADA2-based fungal pipeline by customizing the steps from quality filtering to taxonomic annotation and applied it to patient samples.

## Results

### Preparation of a mock community ITS1 data set.

**Figure 1:**
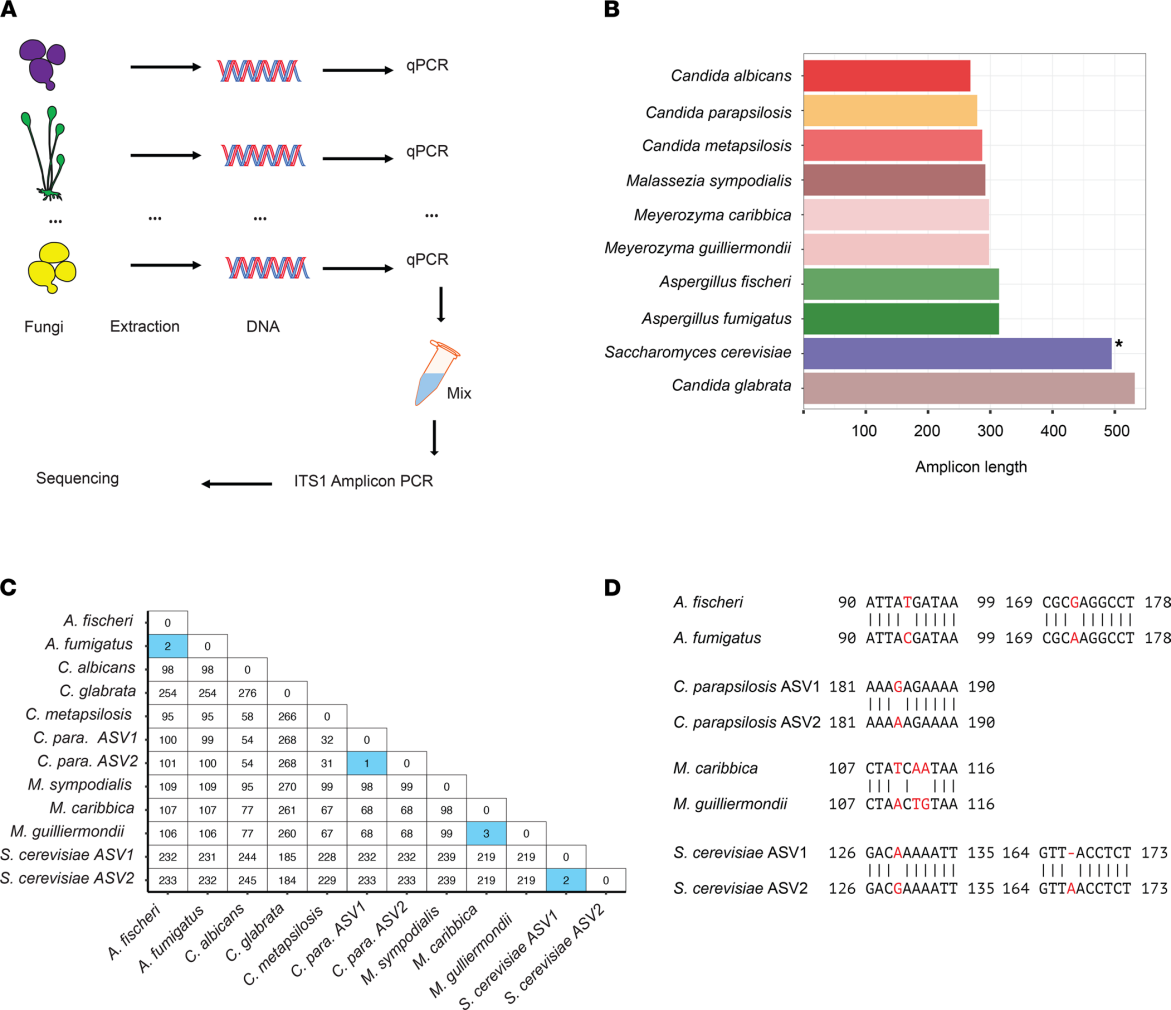
Overview of data set. (**A**) Sample and Sequencing workflow. (**B**) Length variation in the amplified ITS1 region of strains within the mock community. The 2 *S. cerevisiae* ASVs differed by one nucleotide in length. (**C**) Pairwise Levenshtein distance between the expected ITS1 amplicon sequences included in the mock community. (**D**) Nucleotide differences between expected highly similar ITS1 amplicon sequences.

To assess the performance of DADA2 in denoising fungal ITS1 amplicons, we prepared a mock fungal community data set (Figure 1A). We extracted DNA from pure cultures of *Aspergillus fischeri*, *Aspergillus fumigatus*, *Candida albicans*, *Candida glabrata*, *Candida metapsilosis*, *Malassezia sympodialis*, *Meyerozyma caribbica*, *Meyerozyma guilliermondii*, *Saccharomyces cerevisiae*, and 2 strains of *Candida parapsilosis* (Supplemental Table 1; supplemental material available online with this article; https://doi.org/10.1172/jci.insight.151663DS1).

These species and strains were chosen, based on (a) their medical relevance, (b) their difference in ITS amplicon length (Figure 1B), and (c) the high similarity of ITS1 sequences between some of the species included in the mock community (Figure 1C). The ITS1 amplicons of the 2 *C*. *parapsilosis* strains differed by a single nucleotide, the amplicons of *A*. *fischeri* and *A*. *fumigatus* differed by 2 nucleotides, and the amplicons of *M*. *caribbica* and *M*. *guilliermondii* differed by 3 nucleotides (Figure 1D). The *S*. *cerevisiae* strain included in the mock community has an intragenomically heterogeneous ITS1, and the 2 resulting sequencing variants have 2 nucleotide differences. All the highlighted differences between highly similar amplicon pairs represented less than 1% of the total ITS1 amplicon length and, thus, were below the 97% similarity threshold that is commonly chosen for OTU construction.

After DNA extraction, we calculated and normalized the DNA abundance of each input fungal species via quantitative PCR (qPCR; balanced) and created extreme conditions (extreme 1 and extreme 2) for the 2 species pairs with highly similar ITS1 amplicons (*Meyerozyma* and *Aspergillus*) by diluting one of the species 50-fold. We performed an ITS1 amplicon PCR, followed by sequencing on an Illumina MiSeq platform using PE300 settings.

### Denoising with DADA2 allows high-resolution discrimination of fungal ITS1 amplicon data sets.

**Figure 2:**
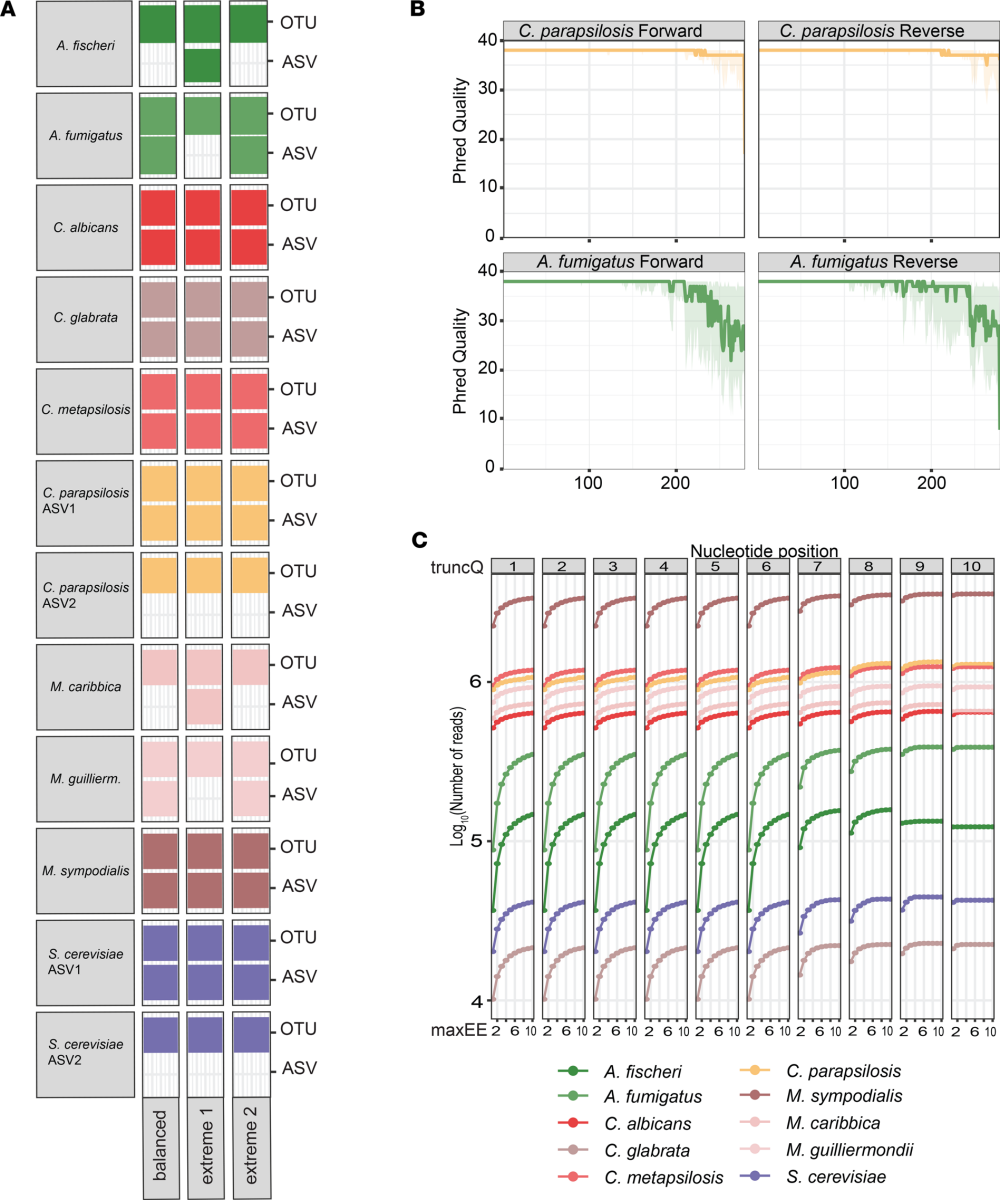
Performance of DADA2 on the mock community data set. (**A**) Strain resolution of DADA2 (ASV) compared with UPARSE (OTU). The balanced community has equal 18S rDNA copy number normalized amounts of DNA per strain. The extreme 1 community include equal 18S rDNA copy number normalized amounts of DNA per strain, except for *A*. *fumigatus* and *M*. *guilliermondii*, which were included at 50-fold dilution. The extreme 2 community include equal 18S rDNA copy number normalized amounts of DNA per strain, except for *A*. *fischeri* and *M*. *caribbica*, which were included at 50-fold dilution. (**B**) Representative quality profile of raw reads that were denoised into exact sequence matches to *A*. *fumigatus* and *C*. *albicans*. The line represents the median Phred score at that position, while the shaded area represents the 25th to 75th percentiles. (**C**) Impact of varying *truncQ* and *maxEE* on the number of species-specific reads.

DADA2 discriminated between individual constituents that differ by a single nucleotide in ITS1 amplicons. In contrast, an OTU-approach (i.e., UPARSE in this example) with a commonly used 97% similarity threshold cannot discriminate these amplicon sequences (Figure 2A). We also confirmed that DADA2 differentiated these species in cases in which one species or strain was highly dominant over another species or strain with a highly similar ITS1 region. DADA2 was able to detect *M*. *guilliermondii* and *M*. *caribbica*, as well as *A*. *fumigatus* and *A*. *fischeri* reads in these extreme conditions. In contrast, an OTU-based approach only resolved the sequence of the amplicon variant with the most reads, since the nucleotide differences in these reads did not pass the 3% dissimilarity threshold to qualify as a distinct OTU (Figure 2A).

### Species-specific bias in ITS1-based amplicon pipelines.

To assess the sequencing quality of raw reads, we developed an R script (https://github.com/thierroll/dada2_custom_fungal/tree/JCI_insight; branch, JCI_insight; commit ID, 380b22dea0596a0c5c09217fbe339a4dbaeae397) that links raw reads to the final ASV assignment. Intriguingly, the read quality differed markedly depending on the fungal species that gave rise to an amplicon (Figure 2B and Supplemental Figure 1). Specifically, reads that originated from the 2 *Aspergillus* species, but also, to a lesser extent, those that originated from *S*. *cerevisiae, C*. *glabrata*, and *M*. *sympodialis* showed a more rapid trail-off of the Phred base quality score (15) toward the 3′ end compared with amplicon sequences that originated from other *Candida* and *Meyerozyma* species. This translated to a high number of expected errors per read (Table 1). These species-specific quality issues did not reflect on the overall quality measures of the sequencing run (Supplemental Table 2).

In the DADA2 workflow, filtering is accomplished by the *filterAndTrim* function and modulated by 2 filtering variables: truncation based on quality scores (*truncQ*) and maximum expected error (*maxEE*). In the manuscript that introduced the concept of *maxEE*, the authors suggested a value of 1, corresponding to no expected error (16). In the DADA2 package, the default value for both *truncQ* and *maxEE* is 2. Individual reads are truncated at the first nucleotide base with a Phred quality score lower than *truncQ*. After truncation, all reads with an equal or higher number of expected errors than the *maxEE* value are removed.

We hypothesized that species-specific differences in the read quality might lead to a bias in the quality filtering step of the DADA2 pipeline. To test this hypothesis, we ran 100 permutations of the 2 filtering variables used in the *filterAndTrim* function, *maxEE* and *truncQ* (Figure 2C).

We confirmed that, by using standard filtering values (*maxEE* = 2 and *truncQ* = 2), most reads pertaining to *Aspergillus* species — and, to a lesser extent, reads pertaining to *S*. *cerevisiae*, *C*. *glabrata,* and *M*. *sympodialis* — would have been discarded. Increasing *maxEE* and *truncQ* values maintained a higher number of reads that belonged to these species by up to 3-fold.

To adjust for species-specific quality differences, the parameters for merging forward and reverse denoised reads could be an alternative variable to modify. Therefore, we assessed whether customizing the *mergePairs* function provides enough or additional benefit compared with customizing the filtering variables. Changing the minimal number of overlapping nucleotides did not affect the number of expected reads that we retrieved, changing the maximum number of mismatches in the overlapping region from 0 to 1 only minimally increased the number of expected reads, and increasing it further had no effect (Supplemental Figures 2 and 3).

### Effect of fine-tuned quality filtering variables on ITS1 data set.

**Figure 3:**
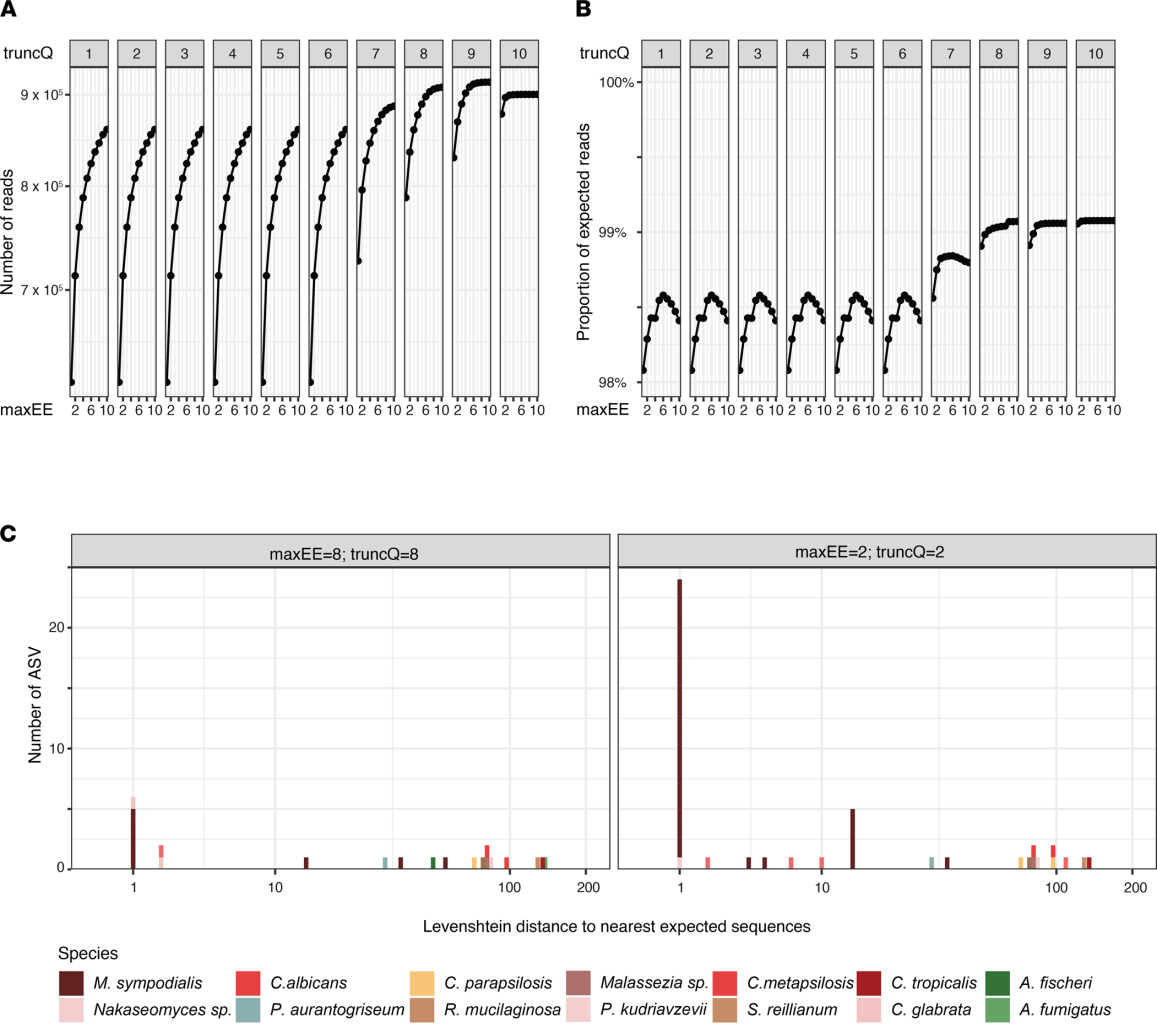
Effect of customizing filtering variables of mock community data set. (**A** and **B**) Number of reads and ration of expected to nonexpected sequences for different combinations of *truncQ* and *maxEE*. (**C**) Comparison of the Levenshtein distance for all nonexpected sequences to the nearest expected ASV reference sequences.

We assessed the impact of customizing the filtering variables on the overall number of retained sequences and on the proportion of expected sequences. The overall number of retained reads increased with higher *maxEE* values and with higher *truncQ* values above a threshold of 6 (Figure 3A). At *truncQ* values below 6, increasing *maxEE* up to 6 increased the proportion of expected reads, while a further increase had a detrimental effect (Figure 3B). Based on these results, we used a value of 8 for both *truncQ* and *maxEE* for further analyses.

With both the customized and default filtering combinations, we retrieved all 12 expected ASVs. The number of nonexpected ASVs (noise) decreased from 46 to 22 in the balanced community when the customized filtering values were used. The decrease in nonexpected ASVs was mainly due to ASVs that differed from the expected sequence by 14 nucleotides or fewer, with the largest reduction seen in ASVs that were assigned to *M*. *sympodialis* (Figure 3C and Supplemental Table 3). With customized filtering variables in place, the overall proportion of noisy reads remained below 1%.

### Effect of filtering customization on real-world data.

**Figure 4:**
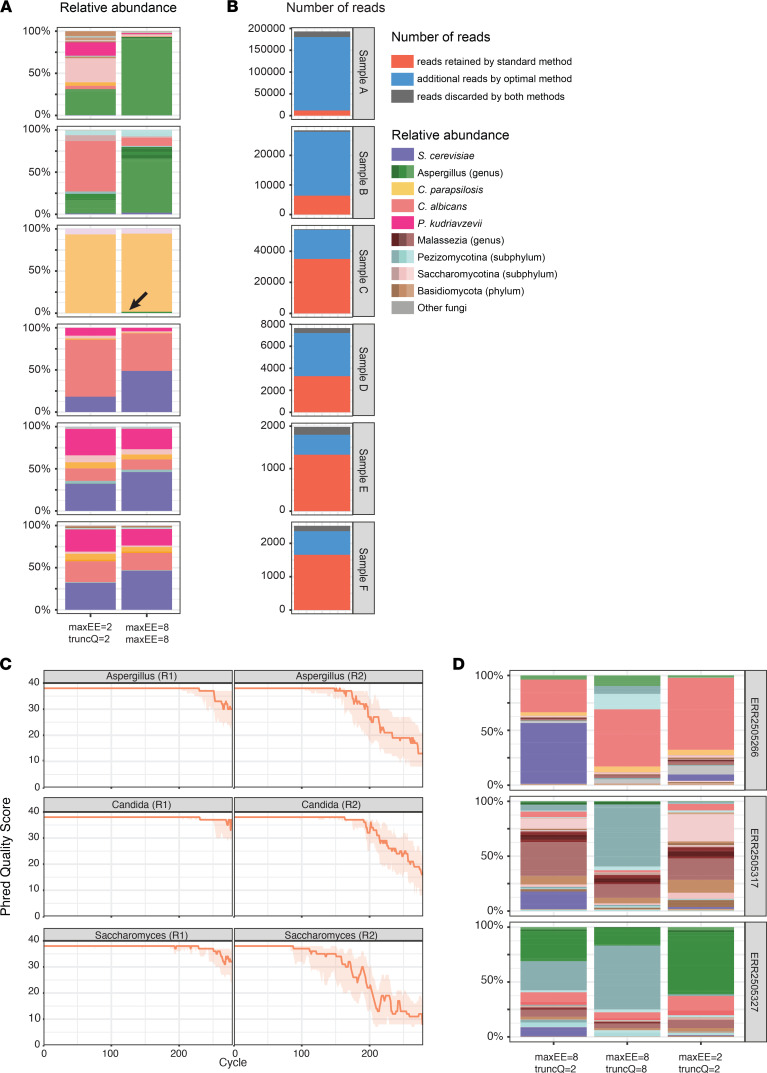
Effect of customizing filtering values on patient samples. (**A**) Taxonomic composition of fecal samples according to the filtering strategy used. The arrow shows the retention of reads from Aspergillus species, which were completely discarded by the standard filtering strategy. (**B**) Number of reads retained by DADA2 according to filtering strategy used. (**C**) Phred quality scores along the R1 and R2 reads of selected fungal genera, generated from ref. 6. (**D**) Taxonomic composition of representative samples from ref. 6 according to the filtering strategy used.

We assessed the effect of customizing the filtering strategy on high-throughput ITS sequencing of patient fecal samples (Figure 4A). We confirmed that the customization enabled us to substantially increase the relative abundance of reads pertaining to *Aspergillus* species (Sample A–Sample C). By using the standard filtering variables, no *Aspergillus* reads were detected in Sample C. The relative abundance of reads pertaining to *S*. *cerevisiae* were also increased with customized filtering (Samples D-F), though to a lesser extent than the *Aspergillus* reads. For both species, the increase in the relative abundance correlated with an increase in the total number of reads that were retained by the customized filtering strategy (Figure 4B). Reads that were discarded by the customized method were evenly discarded across the different steps of the DADA2 pipeline (Supplemental Table 4).

To exclude an effect based on institution-specific protocols, we analyzed the sequencing quality of an external publicly available ITS1 data set (6) (Figure 4, C and D). Importantly, the authors used different ITS1 primers and a different library preparation strategy, resulting in forward reads exclusively in R1 (Illumina first mates) and in reverse reads exclusively in R2 (Illumina second mates). We confirmed taxon-specific quality differences in this data set, with a faster quality trail-off for *Aspergillus* and *Saccharomyces* reads compared with *Candida* reads. As expected, the reverse reads (R2) had a lower quality than the forward reads (R1) due to the library preparation method chosen in this protocol, in which the R1 and R2 adapters were incorporated in the forward and reverse ITS1 primers, respectively. Customizing the filtering variables in the DADA2 pipeline led to changes in the computed taxonomic constitution of individual samples. Importantly, due to the longer *Saccharomyces* ITS1 size and the fast quality trail-off, truncating at a Phred score of 8 led to nonoverlapping sequences and a complete absence of *Saccharomyces* reads. In contrast, modifying only *maxEE* led to an absolute and relative increase of *Saccharomyces* reads and, to some extent, *Aspergillus* reads. These findings emphasize the need to individually customize the DADA2 pipeline to institutional ITS1 protocols.

### Optimizing ITS1 primers for S. cerevisiae and C. glabrata.

**Figure 5:**
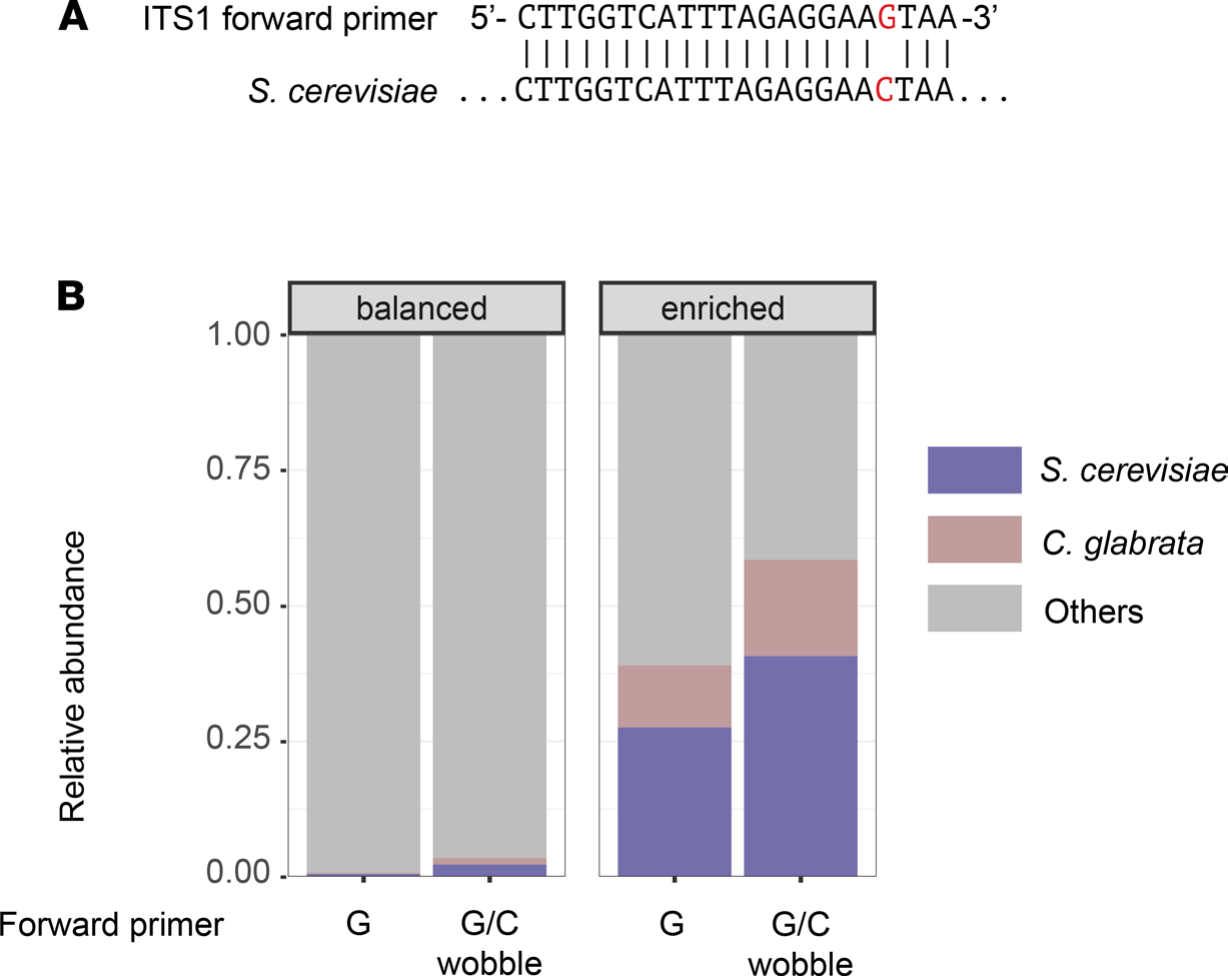
Length-specific biases in fungal ITS1 amplicon sequencing and adaptation of forward primers for better recall of *S. cerevisiae* and *C. glabrata*. (**A**) Single nucleotide difference between the ITS1 forward primer and the *S*. *cerevisiae* reference genome. (**B**) Impact on the relative abundance of *S*. *cerevisiae* and *C*. *glabrata* when using a wobble forward primer allowing for the single nucleotide difference between the ITS1 forward primer and the *S*. *cerevisiae* reference genome.

Returning to the mock community assembled for this study*,* the number of reads attributed to *C*. *glabrata* and *S*. *cerevisiae* were approximately 1 log_10_ lower than the reads of other species (Figure 2A), even though we normalized the amount of input rDNA in the balanced sample and adapted the filtering parameters (17). This result is explained in part by sequencing bias of the Illumina platform against the longer ITS1 amplicon of these 2 species (Figure 1B). Beyond the impact of amplicon length, we hypothesized that a single nucleotide difference in the primer region of the forward primer (ITS1-F) between the reference genome of *S*. *cerevisiae,* some *C*. *glabrata* strains, and other fungal taxa could be responsible for a portion of the observed species-specific bias (Figure 5A). By using an alternative primer with a wobble nucleotide at the diverging position near the 3′ end, the yield of *C*. *glabrata* and *S*. *cerevisiae* reads increased 7.2-fold in the balanced mock community and 2.0-fold in a community maximally enriched in both species (90% of input DNA; Figure 5B).

### Taxonomic annotation for fungal ITS1 amplicon data sets.

**Figure 6:**
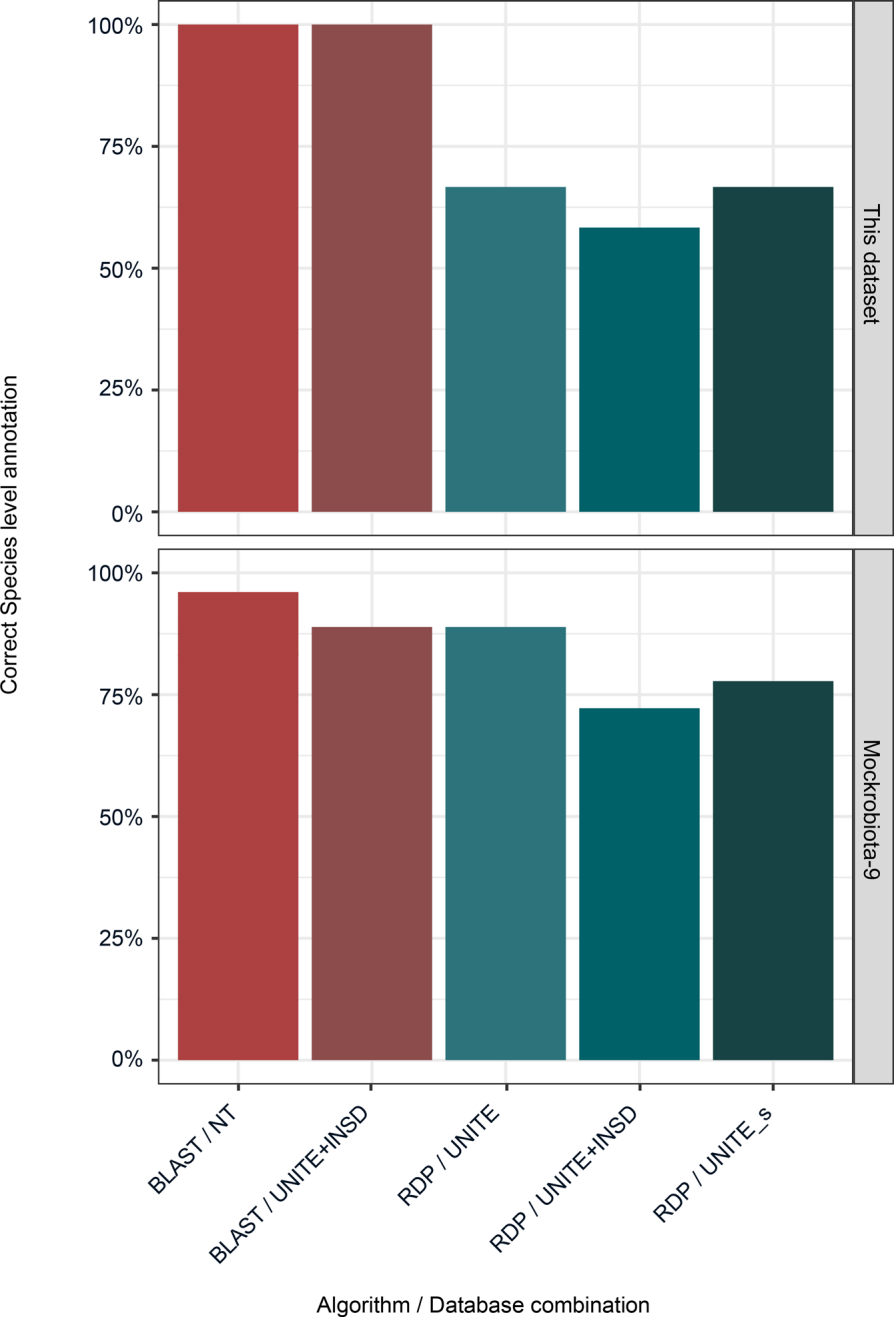
Percentage of correct species-level annotation for different algorithm/database combinations.

Multiple combinations of annotation algorithms and reference databases have been developed for taxonomic annotation. To find an optimal combination, we compared 3 regularly updated reference databases (UNITE with 3 different versions, NCBI NT, and NCBI ITS RefSeq Fungi) and 2 annotation algorithms (RDP and BLAST). DADA2 implements a naive Bayes classifier (RDP classifier) in its *assignTaxonomy* function. The ITS-specific workflow recommends using a UNITE database without specifying which database version to use (https://benjjneb.github.io/dada2/ITS_workflow.html). We tested 3 different versions of the UNITE database: (a) including singletons as reference sequences (UNITE; DOI: 10.15156/BIO/786368), (b) including global and 97% singletons (UNITE_s; DOI: 10.15156/BIO/786369), and (c) the full UNITE+INSD (DOI: 10.15156/BIO/786372) database. All 3 databases resulted in the correct taxonomic annotation at the genus level. With the default bootstrap threshold of 50, correct species-level annotation was not achieved in 4 or 5 of 12 ASVs, depending on the database used (Figure 6 and Supplemental Table 5). For most of these annotations, the uncertainty was acknowledged by not calling a species-level taxonomy. However, sequences for *M*. *carribica* were incorrectly called as *M*. *guilliermondii* for UNITE_s and UNITE, and as *M*. *carnophila* for UNITE+INSD. With the bootstrap threshold set at 80 (suggested for reads longer than 250 nucleotides; ref. 18), the proportion of species-level annotation would decrease even further. Species-level identification was inconsistent between the different versions of the UNITE database. We set a predefined seed in advance to ensure that the naive Bayes classifier algorithm returned reproducible results when rerunning the analyses.

The RDP classifier implemented in the *assignTaxonomy* function does not allow customization to return more than 1 hit. In contrast, the BLAST-based algorithm, as we implemented it, did not return a single, most likely hit, but rather a list of the top potential hits based on E-value or other scores. We arranged these ties based on an available species-level designation and on the number of times a specific species-level designation was returned. With this strategy, both the full UNITE+INSD and the NCBI NT databases allowed a correct species-level annotation for all query sequences (Figure 6 and Supplemental Tables 6 and 7). In contrast, the BLAST-based algorithm was ineffective in returning a correct species-level annotation with the alternative fungal database NCBI ITS_RefSeq_Fungi (Supplemental Table 8).

We tested these combinations of algorithm and database on an external data set (mockrobiota community 9; Mock-9) (19). For these sequences, our BLAST-based algorithm had a similar performance to an RDP-based algorithm (Figure 6 and Supplemental Tables 9–11).

## Discussion

This study demonstrates that a DADA2-based denoising algorithm distinguishes fungal ITS1 amplicon reads that differ by a single nucleotide, as previously demonstrated for bacterial 16S amplicon data sets (7). This discriminatory power allows for species-level distinctions for the members in our mock community of medically relevant fungi. This discriminatory power is not achievable by OTU-based approaches due to grouping clusters of sequences with 97% similarity (20). Additionally, independent DADA2 runs yield the same ASV classification, allowing comparisons between different studies.

The high resolution provides the possibility to identify intraspecies variability, if there is a difference in the ITS1 amplicon, as shown in the discrimination of 2 *C*. *parapsilosis* strains with a single nucleotide polymorphism. This fine discriminatory power was harnessed to track individual *C*. *parapsilosis* strains across different body sites and time to determine the relationship of intestinal and bloodstream isolates in a pathogenesis study (3). In *S*. *cerevisiae* and certain other species, fungal rDNA is present in multiple copies and can contain intragenomic polymorphisms, resulting in the presence of more than 1 ASV for a given clone (21, 22). The optimized DADA2 pipeline can discriminate these polymorphisms and return distinct ASVs for a single clonal origin. On the other end of the spectrum, it is possible that 2 fungal species have an identical ITS1 (23). Thus, it is important to note that fungal ITS1-based ASVs are not a substitute to define a specific fungal species. Diversity measures may be overestimated at the fungal ASV level (24). We feel these limitations are clearly outweighed by the benefit of higher taxonomic resolution associated with an ASV-based approach compared with an OTU-based approach with a 97% similarity threshold. If needed, analysis at higher taxonomic levels remains possible.

Due to the decrease in sequencing quality toward the end of Illumina reads, it is generally recommended to trim reads at the 3′ end in processing bacterial 16S data (7). With this trimming step, the overall quality increases, and more reads can pass the quality filter implemented in the pipeline (25). With the variation in ITS amplicon length, this approach is not generally recommended for fungal data sets. Besides the possibility of trimming reads at a fixed length, the *filterAndTrim* function of the DADA2 package incorporates the possibility to trim reads at the first position with a Phred score lower than a prespecified threshold. By default, this threshold is set to 2. In this study, we showed that increasing this threshold to 8 increased the number of reads that passed the second quality filter step and were denoised correctly.

Quality filtering with the *filterAndTrim* function was performed by removing reads with a higher expected error than a specified threshold. Using the number of expected errors within a read has been shown to be a superior filtering strategy to using the overall or average quality of a read (16). In our data set, we show that increasing the threshold leads to a better recovery of reads that are expected to be present in the sample. Since DADA2 relies on the distribution of sequencing errors, we speculate that including a higher number of erroneous reads may increase the reliability of the error model.

Intriguingly, changing the filtering and trimming parameters affected specific fungal taxa differentially, a phenomenon that has not been described widely in the literature. The ITS regions of different fungal taxa vary considerably in sequence, length, and GC content (26), but this is not likely to influence sequencing quality. An alternative hypothesis would be that DNA extraction techniques affect the DNA of different fungal taxa in different ways. It is critical to consider the impact on differential read quality in the analysis of ITS data sets to minimize any taxon-specific filtering bias.

It is important to highlight that taxon-specific differences vary with the sequencing strategy and the primers used. We demonstrated that changing the parameters of the DADA2 pipeline leads to markedly different results both in an internal and in an external data set. We therefore advise researchers to individually customize DADA2 parameters based on institution-specific protocols and mock data sets to ensure a reliable taxonomic fungal representation. To ensure reproducibility, we encourage researchers to publish the code and relevant pipeline variables together with the results of the analysis.

Besides quality differences of sequences obtained from different species, an additional species-specific bias can be introduced by the commonly used ITS1 forward primer, as it differs by a single nucleotide from the complementary regions for taxa such as *S*. *cerevisiae* and *C*. *glabrata*. The impact of this primer modification is measurable yet moderate in comparison with other biases, such as variations in amplicon length and in rDNA copy numbers between taxa (27). While ITS-based mycobiota analysis will detect different members of fungal communities in high resolution, it can only approximate their relative abundance. However, it remains an extremely valuable tool to classify community members, to assess temporal and spatial variations of the mycobiota, and to monitor exponential expansion of pathogenic fungal taxa seen in specific disease states (3).

Shotgun metagenomics may provide a less biased analysis of microbial communities. However, in most communities, such as the human intestine, the overall abundance of fungi is low. Without the enrichment step inherent to amplicon sequencing, these fungal communities cannot be readily detected in shotgun metagenomic data sets at current sequencing depths. In addition, reference databases for shotgun metagenomic analyses are either absent or incomplete (28). At the present time, ITS-based amplicon approaches remain a cost-efficient standard to profile fungal communities.

Correct genus-level assignment was achievable for ITS1 amplicon data sets either via the RDP naive Bayesian classifier implemented in DADA2 or via a BLAST-based approach, irrespective of the reference database. However, both approaches had limitations. Slight differences in the version of the database gave rise to inconsistent species-level results using the RDP algorithm.

A BLAST-based approach is limited by the fact that the single best hit is not obligately returned, since the algorithm stops after a predefined number of hits have passed the E-value threshold. The RDP algorithm allows acknowledgment of uncertainty only in cases in which the bootstrap value falls below a certain threshold and in which species-level annotation is not called.

In all cases, genus-level annotation was highly accurate irrespective of the chosen algorithm. In studies of the human mycobiota, species-level annotation is desirable due to differing phenotypic characteristics of species within a genus, such as *C*. *albicans* and *C*. *parapsilosis* (29, 30). Species-level annotation is associated with a higher level of uncertainty than genus-level annotation. To confirm biologically meaningful associations, it is therefore advisable to confirm taxonomic annotations by culture-based methods.

In this study, we achieved improved levels of species-level annotation for our community of medically relevant fungi with the BLAST-based algorithm than with the RDP algorithm, and a similar performance for an external data set. It is important to state that the RDP classifier has not been designed specifically for species-level annotation (31). In addition to the RDP classifier implemented in the *assignTaxonomy* function, DADA2 includes the function *assignSpecies*, which aims to unambiguously assign species by exactly matching sequences to a reference. It has been designed specifically for short-read 16S sequences. Of note, *assignSpecies* allows for multiple exact hits, but it has not been tested on fungal data sets so far. Ultimately, the choice of algorithm and associated variables is up to the individual researcher. However, it is important that researchers document and publish this choice to allow for independent interpretation and comparability.

The NCBI NT and the full UNITE+INSD performed equally well. The NCBI ITS RefSeq database did not result in correct taxonomic annotation at the species level. Of note, the downloadable UNITE databases are updated once yearly, while NCBI databases and the linked NCBI taxonomy are updated continuously (14, 32). In the 2020 iteration of UNITE used for this study, the taxonomy for *Candida* strains was not yet updated to reflect the family/genus denominations (*Debaryomycetaceae* as the family for *C*. *albicans*, *C*. *parapsilosis*, and *C*. *metapsilosis*, and *Saccharomycetaceae* as family and *Nakaseomyces* as genus for *C*. *glabrata*). It is important to use the newest version of either database to reflect the rapidly changing fungal taxonomy or to correct nomenclature manually (12, 14).

In summary, we have established that a DADA2 based pipeline can discriminate ITS1 amplicons with single nucleotide resolution as a proof of concept using a representative mock community of medically relevant fungi. While ITS-inherent species-specific biases cannot be overcome fully, customization of a the DADA2-based analytic pipeline can lead to more accurate representation of fungal communities.

## Methods

### Fungal strains and DNA preparation.

We selected 11 different fungal strains from 10 distinct species for analysis (Supplemental Table 11). These strains were chosen to reflect a range of distinct medically relevant fungi and included strains with more than 97% identity in the ITS1 amplicon (*A*. *fumigatus* and *A*. *fischeri*, *M*. *caribbica* and *M*. *guilliermondii*, and 2 *C*. *parapsilosis* strains). Fungal strains were revived from glycerol stock and streaked on YPD agar, cultured at 37°C overnight. Then, the strains were inoculated in YPD liquid medium and cultured overnight at 37°C, shaking at 240 rpm. Fungal cells were harvested and washed twice with sterile water. Fungal DNA was extracted with the QIAamp DNA mini kit (Qiagen 51306).

### Composition of DNA pools.

The 18S copy number per μL of DNA for each strain was measured by qPCR (33). DNA of all the strains was pooled at equal amount of 18S copy numbers for the balanced community. For the extreme 1 community, equal amounts of DNA were pooled for all strains, except for *A*. *fumigatus* and *M*. *guilliermondii*, which were both diluted 50-fold. For the extreme 2 community, equal amounts of DNA were used for all strains, except for *A*. *fischeri* and *M*. *caribbica*, which were both diluted 50-fold. Finally, the enriched community was composed of 10% of the balanced community DNA, 45% of *S*. *cerevisiae* DNA, and 45% *C*. *glabrata* DNA.

### Fecal samples.

Fecal samples were drawn from a fecal biorepository of patients undergoing allogeneic hematopoietic cell transplantation at MSKCC (3, 34). We selected fecal samples from different sequencing runs that contained reads attributed to *Aspergillus*. Samples were processed as described previously (3).

### Amplicon production and sequencing.

We amplified the ITS1 region with the primer set ITS-1-F (5′-CTTGGTCATTTAGAGGAAGTAA-3′) and 5.8S-1R (5′-GTTCAAAGAYTCGATGATTCAC-3′). We also tested an alternative forward primer including a replacement wobble nucleotide (5′-CTTGGTCATTTAGAGGAA**S**TAA-3′). The DNA was amplified for 35 cycles (1 × 98°C, 1 × 53°C, and 1× 72°C, for 30 seconds each) using Phusion polymerase (F530L), as reported previously (3). The ensuing amplicons were sequenced on an Illumina Miseq platform with paired-end 300 setting after library preparation. The amplicon and sequencing strategy result in both forward and reverse reads being present in the R1 and R2 reads. The raw reads were preprocessed by separating forward and reverse reads based on primer presence into 2 different files. Subsequently, primers and (partial) read-ins into the opposite primer were removed by using *cutadapt* (35).

### Denoising and OTU clustering.

Denoising was performed using the DADA2 package in R (7). No fixed length trimming was used. To test different filtering strategies, 100 iterations of the *filterAndTrim* function with *maxEE* and *truncQ* values varying between 1 and 10 each were performed on the preprocessed reads of the balanced community with the ITS-1-F/5.8S-1R primer set. Additionally, we assessed variations on the *minOverlap* and *maxMismatch* variables within the *mergepairs* function. For all other analyses, the ASV object obtained by using *maxEE* and *truncQ* of 8 each was used. OTU clustering was performed via UPARSE by using a customized pipeline based on USEARCH and VSEARCH using the suggested value of *maxEE* of 1 (36–38) and a 97% similarity threshold.

To assess the effect of varying filtering variables on an external data set, we downloaded the ITS1 sequencing data from a study on age-related variations of the microbiota and mycobiota (6). These reads were subsequently processed similarly to sequences from our institution.

### Taxonomic annotation.

To test the RDP naive Bayes classifier implemented in the *assignTaxonomy* function of DADA2, we downloaded 3 variants of the UNITE database, version 8.2 (February 2020) (13). DADA2 can utilize 2 variants of the general FASTA release, one that includes singletons as reference sequences (DOI: 10.15156/BIO/786368) and another that includes global and 97% singletons (DOI: 10.15156/BIO/786368). The third variant consists of the full UNITE+INSD data set (DOI: 10.15156/BIO/786372). The header of this data set was reformatted to comply with DADA2 requirements. We used the default bootstrap threshold of 50 implemented in the DADA2 *assignTaxonomy* function and set the seed of R’s random number generator to 100 for all analyses.

To test a BLAST-based approach to taxonomic assignment, the UNITE+INSD data set was converted to a BLAST-compatible format, NCBI NT and NCBI RefSeq ITS libraries were downloaded in December 2020 from the NCBI FTP site (14, 38, 39). We performed a local BLAST search for the expected sequences with a maximum of 50 target sequences. We calculated the number of times a specific species-level taxonomy was returned per sequence for the NT and the UNITE databases. This was not possible due to the nature of the NCBI ITS database, which includes a unique sequence per species. Additionally, for the UNITE database, we sorted the results on whether a species-level annotation was available or not.

To assess the performance of the taxonomy annotation algorithms on an external data set, we downloaded expected sequences of a fungal mock community (Mock-9) from mock community database *mockrobiota* (19). To allow comparability, we trimmed the expected sequences to cover only the region amplified by our primers. Since 4 of the expected sequences did not include the target of our forward or reverse primers, these were removed from the data set.

### Analysis.

All analyses were performed using R version 4.0.3 (The R Foundation for Statistical Computing).

### Data availability.

Sequences specific to this project have been uploaded to SRA. Code related to the manuscript has been deposited on GitHub: https://github.com/thierroll/dada2_custom_fungal).

### Study approval.

Patients provided written informed consent for biospecimen collection. The fecal biospecimen repository was approved by the MSKCC IRB.

## Author contributions

TR, BZ, TMH, and YT conceived the study. TR and BZ handled sample processing, DNA extraction, and amplicon preparation. TR analyzed the data with assistance by JF and YT. TR wrote the first draft of the manuscript with subsequent contributions by all coauthors. All authors approved the submitted version of the manuscript.

## Supplementary Material

Supplemental data

## Figures and Tables

**Table 1:**
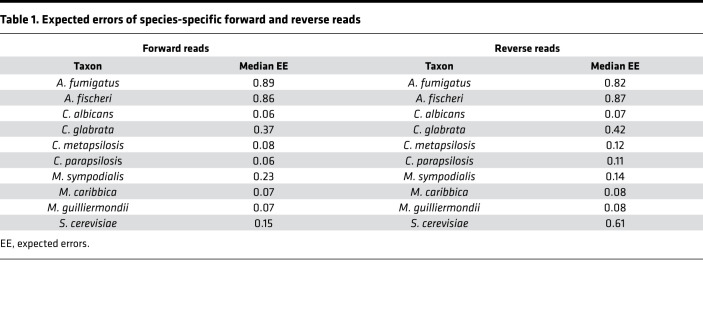
Expected errors of species-specific forward and reverse reads

## Version 1 11/23/2021

In-Press Preview

## Version 2 01/11/2022

Electronic publication
