# Supplementary material for: Customization of a DADA2-based pipeline for fungal internal transcribed spacer 1 (ITS1) amplicon data sets
Source: JCI Insight. 2022 Jan 11;7(1):e151663. doi: 10.1172/jci.insight.151663 (PMC8765055; doi:10.1172/jci.insight.151663)
Supplement: Supplemental data [file jciinsight-7-151663-s076.pdf]

Table S1: Composition of mock community and strain references

Species / Strain	Reference
Candida albicans SC5314	[1]
Candida parapsilosis ASV1	Clinical strain from MSKCC [2]
Candida parapsilosis ASV1	Clinical strain from MSKCC [2]
Meyerozyma caribbica	ATCC 28873™
Meyerozyma guilliermondii	ATCC 6260™
Aspergillus fumigatus AF293	[3]
Aspergillus fischeri	ATCC 1020™
Saccharomyces cerevisiae	Clinical strain from MSKCC
Candida metapsilosis	Clinical strain from MSKCC [2]
Malassezia sympodialis	[4]
Candida glabrata	Clinical strain from MSKCC

1. Ngo, L.Y., et al., *Inflammatory monocytes mediate early and organ-specific innate defense during systemic candidiasis.* J Infect Dis, 2014. **209**(1): p. 109-19.
2. Zhai, B., et al., *High-resolution mycobiota analysis reveals dynamic intestinal translocation preceding invasive candidiasis.* Nat Med, 2020.
3. Hohl, T.M., et al., *Aspergillus fumigatus triggers inflammatory responses by stage-specific beta-glucan display.* PLoS Pathog, 2005. **1**(3): p. e30.
4. Ianiri, G., et al., *Gene Function Analysis in the Ubiquitous Human Commensal and Pathogen Malassezia Genus.* mBio, 2016. **7**(6).

Table S2: Run-specific Illumina Miseq quality measures

Run	Clusters passing filter (%)	Q30 (%)	Error Rate (PhiX)
Mock community	95.16	86.36	2.74
Clinical Sample Run 1	96.99	84.24	3.53
Clinical Sample Run 2	90.00	79.27	3.65
Clinical Sample Run 3	95.43	91.01	2.15
Clinical Sample Run 4	92.54	89.37	2.48

Table S3: Characterization of non-expected sequences in the balanced community compared by filtering status used.

Species (BLAST vs. UNITE+INSD)	Number of sequences	Relative abundance	Levenshstein distance to neares expected sequence	Filter	Sequence
Candida albicans	1263	0.00176968	2	standard	GGAATTCTGCAATTCATATTACGTATCGCATTTCGCTGCGTTCTTCATCGATGCGAGAACCAAGAGATCCGTTGTTGAAAGTTTTGACTATTAGTAATAATCTGGTGTGACAAGTTGATAAAAAATTGGTTGTAAGTTTAGACCTCTGGCGGCAGGCTGGGCCCACCGCCAAAGCAAGTTTGTTTCAAAGAAAAACACATGTGGTGCAATTAAGCAAATCAGTAATGATCCTTCCGCAGGTTCACCTACGGAAACCTTGTTACGACTT
Nakaseomyces sp	34	4.76E-05	1	standard	GGAATTCTGCAATTCACATTACGTATCGCATTTCGCTGCGTTCTTCATCGATGCGAGAACCAAGAGATCCATTGTTGAAAGTTTTGAAGTTGTTTTCTACTAAAAGAAATCTTGTGTTGACTGAATTAGTTTAAAAAAATATTTGTTTGTGTTTGCATCCACTGGGAGAACTCCCCCCCGAAAGAGAGCGTTCCCCCAACGAACAAAAGAATAGTAGTAAAGTAAACTCCACTGTGTGTAGTAATTAGAAAGTGTCGAGTCGTGTGATAAAACACCTCCTTTGGAATAGAGAGATCCACGCACACTCCCAGGTCTTTGTCGGCTCCCTCCCCCCACTGCAGAACACCCACCAACCGCGCACTTAAGCGCAGGCAGGAGAAATAGCATTCACAGCAGAGAAAATATTTTAGGAGCCTCCTGAGTGTCTACACTGGTCCTCCCCAGAGATGTCTCTCTCCGAGCTCAGACAAATCAATTAAATTTCTTT

					AATGATCCTTCCGCAGGTTCACCTACGGAAAC<br>CTTGTTACGACTT
Malassezia sympodialis	479	0.00067116	1	standard	GGAATTCTGCAATTCACATTACCTATCGCGTTT<br>CGCTGCGTTCTTCATCGATGGGAGAACCAAGA<br>GATCCGTTGTCGAAAGTTGTGTATAGTTCGTA<br>GGCTATACCTACAATTTCACGTACATTCAACCA<br>TACGAGTTTTTTTTGTTCGATTACATTCGTCATC<br>ACAGTGCACAGGGGTTGGTATGGATATAGTG<br>ATGGGCGCCAGAGACGTGTTTGCGTCCGTATG<br>GCAGGCCCGAAACTTTCACTAATGATCCTTCTG<br>CAGGTTCACCTACAGAAACCTTGTTACGACTT
Malassezia sympodialis	506	0.00070899	1	standard	GGAATTCTGCAATTCACATTACCTATCGCGTTT<br>CGCTGCGTTCTTCATCGATTGGAGAACCAAGA<br>GATCCGTTGTCGAAAGTTGTGTATAGTTCGTA<br>GGCTATACCTACAATTTCACGTACATTCAACCA<br>TACGAGTTTTTTTTGTTCGATGACATTCGTCAT<br>CACAGTGCACAGGGGTTGGTATGGATATAGTG<br>ATGGGCGCCAGAGACGTGTTTGCGTCCGTATG<br>GCAGGCCCGAAACTTTCACTAATGATCCTTCTG<br>CAGGTTCACCTACAGAAACCTTGTTACGACTT
Malassezia sympodialis	452	0.00063333	1	standard	GGAATTCTGCAATTCACATTACCTATCGCGTTT<br>CGCTGCGTTCTTCATCGATGGGATAACCAAGA<br>GATCCGTTGTCGAAAGTTGTGTATAGTTCGTA<br>GGCTATACCTACAATTTCACGTACATTCAACCA<br>TACGAGTTTTTTTTGTTCGATGACATTCGTCAT<br>CACAGTGCACAGGGGTTGGTATGGATATAGTG<br>ATGGGCGCCAGAGACGTGTTTGCGTCCGTATG<br>GCAGGCCCGAAACTTTCACTAATGATCCTTCTG<br>CAGGTTCACCTACAGAAACCTTGTTACGACTT
Malassezia sympodialis	676	0.00094719	1	standard	GGAATTCTGCAATTCACATTACCTATCGCGTTT<br>CGCTGCGTTCTTCATCGATGGGAGAACCAAGA

					GATCCGTTGTCGAAAGTTGTGTATAGTTAGTAGGCTATACCTACAATTTCACGTACATTCAACCATACGAGTTTTTTTTGTTCGATGACATTCGTCATCACAGTGCACAGGGGTTGGTATGGATATAGTGATGGGCGCCAGAGACGTGTTTGCGTCCGTATGGCAGGCCCGAAACTTTCACTAATGATCCTTCTGCAGGTTCACCTACAGAAACCTTGTTACGACTT
Issatchenkia orientalis	132	0.00018496	84	standard	GATGGCTGCAATTCACACTAGGTATCGCATTTCGCTGCGCTCTTCATCGATGCGAGAACCAAGAGATCCGTTGTTGAAAGTTTTGTTTGTTTTTCGTAGATTTCTCTTGTCGACTATATGCTATATTCCACATTTTAGGTGTTGTTGTTTTCGTTCCGCTCACGCAGTGTAGTACTAAATCACAGTAATGATCCTTCCGCAGGTTCACCTACGGAAACCTTGTTACGACTT
Candida parapsilosis	423	0.0005927	72	standard	GAATATCTGCAATTCATATTACTTATCGCATTTCGCTGCGTTCTTCATCGATGCGAGAACCAAGAGATCCGTTGTTGAAAGTTTTGACTATTAAATAATCGGTTGACATTAAATAAAATTTGGTTGAGTTTAATCTCTGGCAGGCCCGAAACTTTCACTAATGATCCTTCTGCAGGTTCACCTACAGAAACCTTGTTACGACTT
Malassezia sympodialis	495	0.00069358	1	standard	GGAATTCTGCAATTCACATTACCTATCGCGTTTCGCTGCGTTCTTCATCGATGGGAGAACCAAGAGATCCGTTGTCGAAAGTTGTGTATAGTTCGTAGGCTATACCTACAATTTCACGTACATTCAACCATACGAGTTTTTTTTGTTCGATGACATTCGTCATCACAGTGCACAGTGGTTGGTATGGATATAGTGATGGGCGCCAGAGACGTGTTTGCGTCCGTATGGCAGGCCCGAAACTTTCACTAATGATCCTTCTGCAGGTTCACCTACAGAAACCTTGTTACGACTT

Malassezia sympodialis	459	0.00064314	1	standard	GGAATTCTGCAATTCACATTACCTATCGCGTTT<br>CGCTGCGTTCTTCATCGATGGGAGAACCAAGA<br>GATCCGTTGTCGAAAGTTGTGTATAGTTCGTA<br>GGCTATACCTACAATTTCACGTACATTCAACCA<br>TACGAGTTTTTTTTGTTAGATGACATTCGTCAT<br>CACAGTGCACAGGGGTTGGTATGGATATAGTG<br>ATGGGCGCCAGAGACGTGTTTGCGTCCGTATG<br>GCAGGCCCGAAACTTTCACTAATGATCCTTCTG<br>CAGGTTCACCTACAGAAACCTTGTTACGACTT
Malassezia sympodialis	313	0.00043857	1	standard	GGAATTCTGCAATTCACATTACCTATCGCGTTT<br>CGCTGCGTTCTTCATCGATGGGAGAACCAAGA<br>GATCCGTTGTCGAAAGTTGTGTATAGTTCGTA<br>GGCTATACCTACAATTTCACGTACATTCAACCA<br>TACGAGTTTTTTTTGTTCGATGACATTCGTCAT<br>CACAGTGCACAGGTGTTGGTATGGATATAGTG<br>ATGGGCGCCAGAGACGTGTTTGCGTCCGTATG<br>GCAGGCCCGAAACTTTCACTAATGATCCTTCTG<br>CAGGTTCACCTACAGAAACCTTGTTACGACTT
Malassezia sympodialis	535	0.00074963	1	standard	GGAATTCTGCAATTCACATTACCTATCGCGTTT<br>CGCTGCGTTCTTCATCGATGGGAGAACCAAGA<br>GATCCGTTGTCGAAAGTTGTGTATAGTTCGTA<br>GGCTATACCTACAATTTCACGTACATTCAACCA<br>TACGAGTTTTTTTTGTTCGATGACATTCGTCAT<br>CACAGTGCACAGGGGTTGGTATGGATATAGTG<br>ATTGGCGCCAGAGACGTGTTTGCGTCCGTATG<br>GCAGGCCCGAAACTTTCACTAATGATCCTTCTG<br>CAGGTTCACCTACAGAAACCTTGTTACGACTT
Malassezia sympodialis	418	0.00058569	1	standard	GGAATTCTGCAATTCACATTACCTATCGCGTTT<br>CGCTGCGTTCTTCATCGATGGGAGAACCAAGA<br>GATCCGTTGTCGAAAGTTGTGTATAGTTCGTA<br>GGCTATACCTACAATTTCACGTACATTCAACCA

					TACGAGTTTTTTTGTTCGATGACATTCGTCAT<br>CACAGTGCACAGGGGTTGGTATTGATATAGTG<br>ATGGGCGCCAGAGACGTGTTTGCGTCCGTATG<br>GCAGGCCCGAAACTTTCACTAATGATCCTTCTG<br>CAGGTTCACCTACAGAAACCTTGTTACGACTT
Malassezia sympodialis	325	0.00045538	1	standard	GGAATTCTGCAATTCACATTACCTATCGCGTTT<br>CGCTGCGTTCTTCATCGATGGGAGAACCAAGA<br>GATCCGTTGTCGAAAGTTGTGTATAGTTCGTA<br>GGCTATACCTACAATTTCACGTACATTCAACAA<br>TACGAGTTTTTTTGTTCGATGACATTCGTCAT<br>CACAGTGCACAGGGGTTGGTATGGATATAGTG<br>ATGGGCGCCAGAGACGTGTTTGCGTCCGTATG<br>GCAGGCCCGAAACTTTCACTAATGATCCTTCTG<br>CAGGTTCACCTACAGAAACCTTGTTACGACTT
Malassezia sympodialis	417	0.00058429	1	standard	GGAATTCTGCAATTCACATTACCTATCGCGTTT<br>CGCTGCGTTCTTCATCGATGGGAGAACCAAGA<br>GATCCGTTGTCGAAAGTTGTGTATAGTTCGTA<br>GGCTATACCTACAATTTCACGTACATTCAACCA<br>TACTAGTTTTTTTGTTCGATGACATTCGTCATC<br>ACAGTGCACAGGGGTTGGTATGGATATAGTG<br>ATGGGCGCCAGAGACGTGTTTGCGTCCGTATG<br>GCAGGCCCGAAACTTTCACTAATGATCCTTCTG<br>CAGGTTCACCTACAGAAACCTTGTTACGACTT
Malassezia sympodialis	314	0.00043997	1	standard	GGAATTCTGCAATTCACATTACCTATCGCGTTT<br>CGCTGCGTTCTTCATCGATGGGAGAACCAAGA<br>GATCCGTTGTCGAAAGTTGTGTATAGTTCGTA<br>GGCTATACCTACAATTTCACGTACATTCAACCA<br>TACGAGTTTTTTTGTTCGATGACATTCGTCAT<br>CACAGTGCACATGGGTTGGTATGGATATAGTG<br>ATGGGCGCCAGAGACGTGTTTGCGTCCGTATG

					GCAGGCCCGAAACTTTCACTAATGATCCTTCTG CAGGTTCACCTACAGAAACCTTGTTACGACTT
Malassezia sympodialis	315	0.00044137	1	standard	GGAATTCTGCAATTCACATTACCTATCGCGTTT CGCTGCGTTCTTCATCGATGGGAGAACCAAGA GATCCGTTGTCGAAAGTTGTGTATAGTTCGTAT GCTATACCTACAATTTCACGTACATTCAACCAT ACGAGTTTTTTTTGTTCGATGACATTCGTCATC ACAGTGCACAGGGGTTGGTATGGATATAGTG ATGGGCGCCAGAGACGTGTTTGCGTCCGTATG GCAGGCCCGAAACTTTCACTAATGATCCTTCTG CAGGTTCACCTACAGAAACCTTGTTACGACTT
Malassezia sympodialis	327	0.00045818	1	standard	GGAATTCTGCAATTCACATTACCTATCGCGTTT CGCTGCGTTCTTCATCGATGGGAGAACCAAGA GATCAGTTGTCGAAAGTTGTGTATAGTTCGTA GGCTATACCTACAATTTCACGTACATTCAACCA TACGAGTTTTTTTTGTTCGATGACATTCGTCAT CACAGTGCACAGGGGTTGGTATGGATATAGTG ATGGGCGCCAGAGACGTGTTTGCGTCCGTATG GCAGGCCCGAAACTTTCACTAATGATCCTTCTG CAGGTTCACCTACAGAAACCTTGTTACGACTT
Malassezia sympodialis	248	0.00034749	36	standard	GAAAATCTGCAATTCATATTACTTATCGCATTT CGCTGCGTTCTTCATCGATGCGAGAACCAAGA GATCCGTTGTTGAAAGTTTTGAAGATTAATTCA ACCATACGAGTTTTTTTTGTTCGATGACATTCG TCATCACAGTGCACAGGGGTTGGTATGGATAT AGTGATGGGCGCCAGAGACGTGTTTGCGTCCG TATGGCAGGCCCGAAACTTTCACTAATGATCCT TCTGCAGGTTCACCTACAGAAACCTTGTTACGA CTT
Candida albicans	98	0.00013732	10	standard	GAATATCTGCAATTCATATTACTTATCGCATTTC GCTGCGTTCTTCATCGATGCGAGAACCAAGAG

					ATCCGTTGTTGAAAGTTTTGACTATTAAATAATCGGTTGACATTAAATAAAAATTGGTTGTAAGTTTAGACCTCTGGCGGCAGGCTGGGCCCACCGCCAAAGCAAGTTTGTTTCAAAGAAAAACACATGTGGTGCAATTAAGCAAATCAGTAATGATCCTTCCGCAGGTTCACCTACGGAAACCTTGTTACGACTT
Malassezia sympodialis	287	0.00040214	1	standard	GGAATTCTGCAATTCACATTACCTATCGCGTTTCGCTGCGTTCTTCATCGATGGGAGAACCAAGAGATCCGTTGTCGAAAGTTGTGTATAGTTCGTAGGCTATACCTACAATTTCACGTACATTCAACCATACGAGTTTTTTTTGTTCGATGACATTCGTCATCACAGTGCACAGGGGTTGGTATGGATATAGTGATGGGCGCCATAGACGTGTTTGCGTCCGTATGGCAGGCCCGAAACTTTCACTAATGATCCTTCTGCAGGTTCACCTACAGAAACCTTGTTACGACTT
Malassezia sympodialis	427	0.0005983	1	standard	GGAATTCTGCAATTCACATTACCTATCGCGTTTCGCTGCGTTCTTCATCGATGGGAGAACCAAGAGATCCGTTGTCGAAAGTTGTGTATAGTTCGTAGGCTATACCTACAATTTCACGTACATTCAACCATACGAGTTTTTTTTGTTCGATGACATTCGTCATCACAGTGCACAGGGGTTGGTATGGATATAGTGATGGGCGCCAGAGACGTGTTTGCGTCAGTATGGCAGGCCCGAAACTTTCACTAATGATCCTTCTGCAGGTTCACCTACAGAAACCTTGTTACGACTT
Malassezia sympodialis	224	0.00031386	1	standard	GGAATTCTGCAATTCACATTACCTATCGCGTTTCGCTGCGTTCTTCATCGATGGGAGAACCAAGAGATCCGTTGTCGAAAGTTGTGTATAGTTCGTAGGCTATACCTACAATTTCACGTACATTAAACCATACGAGTTTTTTTTGTTCGATGACATTCGTCATCACAGTGCACAGGGGTTGGTATGGATATAGTG

					ATGGGCGCCAGAGACGTGTTTGCGTCCGTATG GCAGGCCCGAAACTTTCACTAATGATCCTTCTG CAGGTTCACCTACAGAAACCTTGTTACGACTT
Malassezia sympodialis	372	0.00052124	1	standard	GGAATTCTGCAATTCACATTACCTATCGCGTTT CGCTGCGTTCTTCATCGATGGGAGAACCAAGA GATCCGTTGTCGAAAGTTGTGTATAGTTCGTA GGCTATACCTACAATTTCACGTACATTCAACCA TACGAGTTTTTTTTGTTCGATGACATTCGTCAT CACAGTGCACAGGGGTTGGTATGGATATAGTG ATGGGCGCCAGAGACGTGTTTGCGTCCGTATG GCAGGCCCGAAACTTTCACTAATGATCATTCTG CAGGTTCACCTACAGAAACCTTGTTACGACTT
Malassezia sympodialis	187	0.00026202	1	standard	GGAATTCTGCAATTCACATTACCTATCGCGTTT CGCTGCGTTCTTCATCGATGGGAGAACCAAGA GATCCGTTGTCGAAAGTTGTGTATAGTTCGTA GGCTATACATACAATTTCACGTACATTCAACCA TACGAGTTTTTTTTGTTCGATGACATTCGTCAT CACAGTGCACAGGGGTTGGTATGGATATAGTG ATGGGCGCCAGAGACGTGTTTGCGTCCGTATG GCAGGCCCGAAACTTTCACTAATGATCCTTCTG CAGGTTCACCTACAGAAACCTTGTTACGACTT
Malassezia sympodialis	190	0.00026622	1	standard	GGAATTCTGCAATTCACATTACCTATCGCGTTT CGCTGCGTTCTTCATCGATGGGAGAACCAAGA GATCCGTTGTCGAAAGTTGTGTATAGTTCGTA GGCTATACCTACAATTTCACGTACATTCAACCA TACGAGTTTTTTTTGTTCGATGACATTAGTCAT CACAGTGCACAGGGGTTGGTATGGATATAGTG ATGGGCGCCAGAGACGTGTTTGCGTCCGTATG GCAGGCCCGAAACTTTCACTAATGATCCTTCTG CAGGTTCACCTACAGAAACCTTGTTACGACTT

Malassezia sympodialis	304	0.00042596	1	standard	GGAATTCTGCAATTCACATTACCTATCGCGTTT<br>CGCTGCGTTCTTCATCGATGGGAGAACAAAGA<br>GATCCGTTGTCGAAAGTTGTGTATAGTTCGTA<br>GGCTATACCTACAATTTCACGTACATTCAACCA<br>TACGAGTTTTTTTTGTTCGATGACATTCGTCAT<br>CACAGTGCACAGGGGTTGGTATGGATATAGTG<br>ATGGGCGCCAGAGACGTGTTTGCGTCCGTATG<br>GCAGGCCCGAAACTTTCACTAATGATCCTTCTG<br>CAGGTTCACCTACAGAAACCTTGTTACGACTT
Malassezia sympodialis	301	0.00042175	1	standard	GGAATTCTGCAATTCACATTACCTATCGCGTTT<br>CGCTGCGTTCTTCATCGATGGGAGAACCAAGA<br>GATCCGTTGTAGAAAGTTGTGTATAGTTCGTA<br>GGCTATACCTACAATTTCACGTACATTCAACCA<br>TACGAGTTTTTTTTGTTCGATGACATTCGTCAT<br>CACAGTGCACAGGGGTTGGTATGGATATAGTG<br>ATGGGCGCCAGAGACGTGTTTGCGTCCGTATG<br>GCAGGCCCGAAACTTTCACTAATGATCCTTCTG<br>CAGGTTCACCTACAGAAACCTTGTTACGACTT
Malassezia sympodialis	284	0.00039793	1	standard	GGAATTCTGCAATTCACATTACCTATCGCGTTT<br>CGCTGCGTTCTTCATCGATGGGAGAACCAAGA<br>GATCCGTTGTCGAAAGTTGTGTATAGTTCGTA<br>GGCTATACCTACAATTTCACGTACATTCAACCA<br>TACGAGTTTTTTTTGTTCGATGACATTCGTCAT<br>CACAGTGCACAGGGGTTGGTATGGATATAGTG<br>ATGGGCGCCAGAGACGTGTTTGCGTACGTATG<br>GCAGGCCCGAAACTTTCACTAATGATCCTTCTG<br>CAGGTTCACCTACAGAAACCTTGTTACGACTT
Malassezia sp	33	4.62E-05	81	standard	GGAATTCTGCAATTCACATTACCTATCGCGTTT<br>CGCTGCGTTCTTCATCGATGGGAGAACCAAGA<br>GATCCGTTGTCGAAAGTTGTGTATAGTTTGTTG<br>GCCAGTGACGGTCCAACCAAGTTCCACGTTCA

					TACTAACCATTCGAGTTTGTAGCGAAGAAAGACCCCAAGAGAGGCCACAAAAAACTTCTCTGAAGCCTTTCTCCAAGACAGTGCACACGGGTTTGTGGATGTATAGTGGGTGTAGGCGGCCAGGGACACTTTTTGGCGTCCGTATGGCCTGCCCAAATCTTCACTAATGATCCTTCTGCAGGTTCACCTACAGAAACCTTGTTACGACTT
Candida metapsilosis	17	2.38E-05	81	standard	GAATATCTGCAATTCATATTACTTATCGCATTTCGCTGCGTTCTTCATCGATGCGAGAACCAAGAGATCCGTTGTTGAAAGTTTTGACTATTAGTTAATCAAGTTGACAATTAATAAAAAATCCATTTTCAAAATTATTAAATTTCTTTAATGATCCTTCCGCAGGTTCACCTACGGAAACCTTGTTACGACTT
Candida albicans	8	1.12E-05	7	standard	GAATATCTGCAATTCATATTACGTATCGCATTTCGCTGCGTTCTTCATCGATGCGAGAACCAAGAGATCCGTTGTTGAAAGTTTTGACTATTAGTAATAATCTGGTGTGACAAGTTGATAAAAAATTGGTTGTAAGTTTAGACCTCTGGCGGCAGGCTGGGCCCACCGCCAAAGCAAGTTTGTTTCAAAGAAAAACACATGTGGTGCAATTATTAAATTTCTTTAATGATCCTTCCGCAGGTTCACCTACGGAAACCTTGTTACGACTT
Penicillium aurantiogriseum	7	9.81E-06	31	standard	TGAATTTGCAATTCACATTACGTATCGCATTTCGCTGCGTTCTTCATCGATGCCGGAACCAAGAGATCCGTTGTTGAAAGTTTTAAATAATTTATATTTTCACTCAGACTTCAATCTTCAGACAGAGTTCGAGGGTGTCTTCGGCGGGCGCGGGCCCGGGGGCGTGAGCCCCCCCGGCGGCCAGTTAAGGCGGGCCCGCCGAAGCAACAAGGTAAAATAAACACGGGTGGGAGGTTGGACCCAGAGGGCCCTCACT

					CGGTAATGATCCTTCCGCAGGTTCACCTACGG AAACCTTGTTACGACTT
Candida metapsilosis	23	3.22E-05	97	standard	GAATATCTGCAATTCATATTACTTATCGCATTTC GCTGCGTTCTTCATCGATGCGAGAACCAAGAG ATCCGTTGTTGAAAGTTTTGACTATTAGTTAAT CAAGTTGACAATTAATAAAAAATTTGGTTGAG TTTAATCTCTGGCAGGCCGTGGGCCCACCAAA GCAAAGTTTTTATTAATTGTCAACTTGATTAAC TAATAGTCAAAACTTTCAACCATACGAGTTTTT TTTGTTCGATGACATTCGTCATCACAGTGCACA GGGGTTGGTATGGATATAGTGATGGGCGCCA GAGACGTGTTTGCGTCCGTATGGCAGGCCCGA AACTTTCACTAATGATCCTTCTGCAGGTTCACC TACAGAAACCTTGTTACGACTT
Rhodotorula mucilaginosa	13	1.82E-05	78	standard	TGAATTCTGCAATTCACATTACTTATCGCATTTC GCTGCGTTCTTCATCGATGCGAGAGCCAAGAG ATCCGTTGTTGAAAGTTTTATTTTGTTATAAAA TTTAATACATTCATAGACTTTGTGTTTATAAGT GAATAGGAGTTCGCTCTCTTGCGAGAGTTACT ATCCCAAACAAGTGCACAGGGTTAGAAAGTGA GAGTTCGGACTCCAAGTTAAGTTGGACGTCCT ATATTCACTAATGATCCTTCCGCAGGTTCACCT ACGGAAACCTTGTTACGACTT
Malassezia sympodialis	13	1.82E-05	4	standard	GGAATTCTGCAATTAACATTACCTATCGCGTTT AGCTGCGTTCTTCATCGATGGGAGAACCAAGA GATCCGTTGTCGAAAGTTGTGTATAGTTAGTA GGCTATACCTACAATTTCACGTACATTAAACCA TACGAGTTTTTTTTGTTCGATGACATTCGTCAT CACAGTGCACAGGGGTTGGTATGGATATAGTG ATGGGCGCCAGAGACGTGTTTGCGTCCGTATG

					GCAGGCCCGAAACTTTCACTAATGATCCTTCTG CAGGTTCACCTACAGAAACCTTGTTACGACTT
Candida parapsilosis	11	1.54E-05	97	standard	GAATATCTGCAATTCATATTACTTATCGCATTTC GCTGCGTTCTTCATCGATGCGAGAACCAAGAG ATCCGTTGTTGAAAGTTTTGACTATTAAATAAT CGGTTGACATTAAATAAAATTTGGTTGAGTTTA ATCTCTGGCAGGCCCCATATAGAAGGCCTACC AAAGCAAAGTTTATTTAATGTCAACCGATTATT TAATAGTCAAAACTTTCAACCATACGAGTTTTT TTTGTTCGATGACATTCGTCATCACAGTGCACA GGGGTTGGTATGGATATAGTGATGGGCGCCA GAGACGTGTTTGCGTCCGTATGGCAGGCCCGA AACTTTCACTAATGATCCTTCTGCAGGTTCACC TACAGAAACCTTGTTACGACTT
Malassezia sympodialis	9	1.26E-05	5	standard	GGAATTCTGCAATTAACATTACCTATCGCGTTT AGCTGCGTTCTTCATCGATGGGAGAACCAAGA GATCCGTTGTCGAAAGTTGTGTATAGTTAGTA GGCTATACCTACAATTTCACGTACATTAAACCA TACGAGTTTTTTTGTTCGATGACATTCGTCAT CACAGTGCACAGGGGTTGGTATGGATATAGTG ATGGGCGCCAGAGACGTGTTTGCGTCAGTATG GCAGGCCCGAAACTTTCACTAATGATCCTTCTG CAGGTTCACCTACAGAAACCTTGTTACGACTT
Sporisorium reilianum	9	1.26E-05	129	standard	TTCTGCAATTCACATTACTTATCGCAATTCGCT GCGTTCTTCATCGATGGGAGAACCAAGAGATC CGTTGCCAAAAGTTGTTTTTAAGATTTTTAGAC GACCGCATTACCAGCCGTAAAAATAATAAATT CTTTCTTAATCCTAGATCATCAAAAGTGTTTTTG TAAGGTGTCAGGTCGTCGTAAAGACACACTCT CGCATGCCTCTACTTCGAACCCGACCGATGCA ACCGTGTTGAAAAAAGGTAGCCCTAGTTTAGT

					TGGACAGGTGCGGGCCGCACCTCAGAAAAAAAAGGTTTTTCATCGAAATGATCCATCTGCAGGTTCACCTACAGATACCTTGTTACGACTT
Candida tropicalis	8	1.12E-05	135	standard	GAATATCTGCAATTCATATTACGTATCGCATTTCGCTGCGTTCTTCATCGATGCGAGAACCAAGAGATCCGTTGTTGAAACTTTCACTAATGATCCTTCTGCAGGTTCACCTACAGAAACCTTGTTACGACTT
Malassezia sympodialis	1	1.40E-06	14	standard	GGAATTCTGCAATTCACATTACCTATCGCGTTTCGCTGCGTTCTTCATCGATGGGAGAACCAAGAGATCCGTTGTCGAAAGTTGTGTATAGTTCGTAGGCTATACCTACAATTTCACGTACATTCAACCATACGAGTTTTTTTGTTCGATGACATTCGTCATCACAGTGCACAGTGGTTGGTATGGATATAGTGATGGGCGCCAGAGACGTGTTTGCGTCCGTATGGCAGGCCCGAAACTTTCACTAATGATCCTTCTGCAGGTTCACCTACAGAAAC
Candida albicans	3	4.20E-06	109	standard	GAATATCTGCAATTCATATTACGTATCGCATTTCGCTGCGTTCTTCATCGATGCGAGAACCAAGAGATCCGTTGTTGAAAGTTTTGACTATTAGTAATAATCTGGTGTGACAAGTTGATAAAAAATTGGTTGTAAGTTTAGACCTCTGGCGGCAGGCTGGGCCCACCGCCAAAGCAAGTTTGTTTCAAAGAAAAACACCAGATTATTACTAATAGTCAAAACTTTCAACCATACGAGTTTTTTTTGTTCGATGACATTCGTCATCACAGTGCACAGGGGTTGGTATGGATATAGTGATGGGCGCCAGAGACGTGTTTGCGTCCGTATGGCAGGCCCGAAACTTTCACTAATGATCCTTCTGCAGGTTCACCTACAGAAACCTTGTTACGACTT

Malassezia sympodialis	1	1.40E-06	14	standard	GGAATTCTGCAATTCACATTACCTATCGCGTTT<br>CGCTGCGTTCTTCATCGATGGGAGAACCAAGA<br>GATCCGTTGTCGAAAGTTGTGTATAGTTCGTA<br>GGCTATACCTACAATTTCACGTACATTCAACCA<br>TACGAGTTTTTTTTGTTCGATGACATTCGTCAT<br>CACAGTGCACAGGGGTTGGTATGGATATAGTG<br>ATGGGCGCCAGAGACGTGTTTGCGTACGTATG<br>GCAGGCCCGAAACTTTCACTAATGATCCTTCTG<br>CAGGTTCACCTACAGAAAC
Malassezia sympodialis	1	1.40E-06	14	standard	GGAATTCTGCAATTCACATTACCTATCGCGTTT<br>CGCTGCGTTCTTCATCGATGGGAGAACCAAGA<br>GATCCGTTGTCGAAAGTTGTGTATAGTTCGTA<br>GGCTATACCTACAATTTCACGTACATTCAACCA<br>TACGAGTTTTTTTTGTTCGATGACATTCGTCAT<br>CACAGTGCACAGGGGTTGGTATGGATATAGTG<br>ATGGGCGCCAGAGACGTGTTTGCGTCCGTATG<br>GCAGGCCCGAAACTTTCACTAATGATCATTCTG<br>CAGGTTCACCTACAGAAAC
Malassezia sympodialis	1	1.40E-06	14	standard	GGAATTCTGCAATTCACATTACCTATCGCGTTT<br>CGCTGCGTTCTTCATCGATGGGAGAACCAAGA<br>GATCCGTTGTCGAAAGTTGTGTATAGTTCGTA<br>GGCTATACCTACAATTTCACGTACATTCAACCA<br>TACGAGTTTTTTTTGTTCGATGACATTCGTCAT<br>CACAGTGCACAGGGGTTGGTATGGATATAGTG<br>ATTGGCGCCAGAGACGTGTTTGCGTCCGTATG<br>GCAGGCCCGAAACTTTCACTAATGATCCTTCTG<br>CAGGTTCACCTACAGAAAC
Malassezia sympodialis	1	1.40E-06	14	standard	GGAATTCTGCAATTCACATTACCTATCGCGTTT<br>CGCTGCGTTCTTCATCGATGGGAGAACCAAGA<br>GATCCGTTGTCGAAAGTTGTGTATAGTTCGTA<br>GGCTATACCTACAATTTCACGTACATTCAACCA

					TACGAGTTTTTTTTGTTCGATGACATTCGTCAT CACAGTGCACAGGGGTTGGTATTGATATAGTG ATGGGCGCCAGAGACGTGTTTGCGTCCGTATG GCAGGCCCGAAACTTTCACTAATGATCCTTCTG CAGGTTCACCTACAGAAAC
Candida glabrata	17	1.87E-05	2	optimal	GGAATTCTGCAATTCACATTACGTATCGCATTT CGCTGCGTTCTTCATCGATGCGAGAACCAAGA GATCCATTGTTGAAAGTTTTGAAGTTGTTTTCT ACTAAAAGAAATCTTGTGTTGACTGAATTAGTT TAAAAAAATATTTGTTTGTGTTTGCATCCACTG GGAGAACTCCCCCCCGAAAGAGAGCTTTCCCC CAACGAACAAAAGAATAGTAGTAAAGTAAACT CCACTGTGTGTAGTAATTAGAAAGTGTCGAGT CGTGTGATAAAACACCTCCTTTGGAATAGAGA GATCCACGCACACTCCCAGGTCTTTGTCGGCTC CCTCCCCCCACTGCAGAACACCCACCAACCGC GCACTTAAGCGCAGGCAGGAGAAATAGCATTC ACAGCAGAGAAAATATTTTAGGAGCCTCCTGA GTGTCTACACTGGTCCTCCCCAGAGATGTCTCT CTCCGAGCTCAGACAAATCAATTAAATTTCTTT AATGATCCTTCTGCAGGTTCACCTACAGAAACC TTGTTACGACTT
Malassezia sympodialis	10	1.10E-05	55	optimal	GAATATCTGCAATTCATATTACTTATCGCATTTC GCTGCGTTCTTCATCGATGCGAGAACCAAGAG ATCCGTTGTTGAAAGTTTTGACTATTAAATAAT CGGTTGACATTAAATAAAATTTGGTTGAGTTTA ATGTCAACCGATTATTTAATAGTCAAAACTTTC AACCATACGAGTTTTTTTTGTTCGATGACATTC GTCATCACAGTGCACAGGGGTTGGTATGGATA TAGTGATGGGCGCCAGAGACGTGTTTGCGTCC GTATGGCAGGCCCGAAACTTTCACTAATGATC

					CTTCTGCAGGTTCACCTACAGAAACCTTGTTAC<br>GACTT
Sporisorium reilianum	14	1.54E-05	129	optimal	TTCTGCAATTCACATTACTTATCGCAATTCGCT<br>GCGTTCTTCATCGATGGGAGAACCAAGAGATC<br>CGTTGCCAAAAGTTGTTTTTAAGATTTTTAGAC<br>GACCGCATTACCAGCCGTAAAAATAATAAATT<br>CTTTCTTAATCCTAGATCATCAAAAGTGTTTTTG<br>TAAGGTGTCAGGTCGTCGTAAAGACACACTCT<br>CGCATGCCTCTACTTCGAACCCGACCGATGCA<br>ACCGTGTTGAAAAAAGGTAGCCCTAGTTTAGT<br>TGGACAGGTGCGGGCCGCACCTCAGAAAAAA<br>AAAGGTTTTTTCATCGAAATGATCCATCTGCAG<br>GTTCACCTACAGATACCTTGTTACGACTT
Aspergillus fumigatus	11	1.21E-05	138	optimal	TGAATTCTGCAATTCACATTACTTATCGCATTTC<br>GCTGCGTTCTTCATCGATGCCGGAACCAAGAG<br>ATCCGTTGTTGAAAGTTTTAACTGATTACGATA<br>ATCAACTCAGACTGCATACTTTCAGAACAGCGT<br>TCATGTTGGGGTCTTCGGCGGGCGCGGGCCC<br>GGGGGCGCAAGGCCTCCCCGGCGGCCGTCGA<br>AACGGCGGGCCCGCCGAAGACCCCAACATGA<br>ACGCTGTTCTGAAAGTATGCAGTCTGAGTTGA<br>TTATCGTAATCAGTTAAAACTTTCAACCATACG<br>AGTTTTTTTTGTTCGATGACATTCGTCATCACA<br>GTGCACAGGGGTTGGTATGGATATAGTGATG<br>GGCGCCAGAGACGTGTTTGCGTCCGTATGGCA<br>GGCCCGAAACTTTCACTAATGATCCTTCTGCAG<br>GTTCACCTACAGAAACCTTGTTACGACTT
Issatchenkia orientalis	143	0.00015691	84	optimal	GATGGCTGCAATTCACACTAGGTATCGCATTTC<br>GCTGCGCTCTTCATCGATGCGAGAACCAAGAG<br>ATCCGTTGTTGAAAGTTTTGTTTGTTTTTCGTA<br>GATTTCTCTTGTCGACTATATGCTATATTCCACA

					TTTTAGGTGTTGTTGTTTTCGTTCCGCTCACGCAGTGTAGTACTAAATCACAGTAATGATCCTTCCGCAGGTTCACCTACGGAAACCTTGTTACGACTT
Candida tropicalis	8	8.78E-06	135	optimal	GAATATCTGCAATTCATATTACGTATCGCATTTCGCTGCGTTCTTCATCGATGCGAGAACCAAGAGATCCGTTGTTGAAACTTTCACTAATGATCCTTCTGCAGGTTCACCTACAGAAACCTTGTTACGACTT
Aspergillus fischeri	6	6.58E-06	49	optimal	TGAATTCTGCAATTCACATTACTTATCGCATTTCGCTGCGTTCTTCATCGATGCCGGAACCAAGAGATCCGTTGTTGAAAGTTTTAACTGATTACGATAATCAACTCAGACTGCATACTTTCAGAACAGCGTTCATGTTGGGGTCTTCGGCGGGCCCGCCGAAGCAACAAGGTACGATAGACACGGGTGGGAGGTTGGACCCAGAGGGCCCTCACTCGGTAATGATCCTTCCGCAGGTTCACCTACGGAAACCTTGTTACGACTT
Candida glabrata	50	5.49E-05	1	optimal	GGAATTCTGCAATTCACATTACGTATCGCATTTCGCTGCGTTCTTCATCGATGCGAGAACCAAGAGATCCATTGTTGAAAGTTTTGAAGTTGTTTTCTACTAAAAGAAATCTTGTGTTGACTGAATTAGTTTAAAAAAATATTTGTTTGTGTTTGCATCCACTGGGAGAACTCCCCCCCGAAAGAGAGCGTTCCCCCAACGAACAAAAGAATAGTAGTAAAGTAAACTCCACTGTGTGTAGTAATTAGAAAGTGTCGAGTCGTGTGATAAAACACCTCCTTTGGAATAGAGAGATCCACGCACACTCCCAGGTCTTTGTCGGCTCCCTCCCCCCACTGCAGAACACCCACCAACCGCGCACTTAAGCGCAGGCAGGAGAAATAGCATTCACAGCAGAGAAAATATTTTAGGAGCCTCCTGAGTGTCTACACTGGTCCTCCCCAGAGATGTCTCT

					CTCCGAGCTCAGACAAATCAATTAAATTTCTTTAATGATCCTTCCGCAGGTTCACCTACGGAAACCTTGTTACGACTT
Malassezia sympodialis	1	1.10E-06	14	optimal	GGAATTCTGCAATTCACATTACCTATCGCGTTTCGCTGCGTTCTTCATCGATGGGAGAACCAAGAGATCCGTTGTCGAAAGTTGTGTATAGTTCGTAGGCTATACCTACAATTTCACGTACATTCAACCATACGAGTTTTTTTTGTTCGATGACATTCGTCATCACAGTGCACAGGGGTTGGTATGGATATAGTGATTGGCGCCAGAGACGTGTTTGCGTCCGTATGGCAGGCCCGAAACTTTCACTAATGATCCTTCTGCAGGTTCACCTACAGAAAC
Candida albicans	1217	0.0013354	2	optimal	GGAATTCTGCAATTCATATTACGTATCGCATTTCGCTGCGTTCTTCATCGATGCGAGAACCAAGAGATCCGTTGTTGAAAGTTTTGACTATTAGTAATAATCTGGTGTGACAAGTTGATAAAAAATTGGTTGTAAGTTTAGACCTCTGGCGGCAGGCTGGGCCCACCGCCAAAGCAAGTTTGTTTCAAAGAAAAACACATGTGGTGCAATTAAGCAAATCAGTAATGATCCTTCCGCAGGTTCACCTACGGAAACCTTGTTACGACTT
Rhodotorula mucilaginosa	14	1.54E-05	78	optimal	TGAATTCTGCAATTCACATTACTTATCGCATTTCGCTGCGTTCTTCATCGATGCGAGAGCCAAGAGATCCGTTGTTGAAAGTTTTATTTTGTTATAAAATTTAATACATTCATAGACTTTGTGTTTATAAGTGAATAGGAGTTCGCTCTCTTGCGAGAGTTACTATCCCAAACAAGTGCACAGGGTTAGAAAGTGAGAGTTCGGACTCCAAGTTAAGTTGGACGTCCTATATTCACTAATGATCCTTCCGCAGGTTCACCTACGGAAACCTTGTTACGACTT

Malassezia restricta	48	5.27E-05	81	optimal	GGAATTCTGCAATTCACATTACCTATCGCGTTTCGCTGCGTTCTTCATCGATGGGAGAACCAAGAGATCCGTTGTCGAAAGTTGTGTATAGTTTGTTGGCCAGTGACGGTCCAACCAAGTTCCACGTTCATACTAACCATTCGAGTTTGTAGCGAAGAAAGACCCCAAGAGAGGCCACAAAAAACTTCTCTGAAGCCTTTCTCCAAGACAGTGCACACGGGTTTGTGGATGTATAGTGGGTGTAGGCGGCCAGGGACACTTTTTGGCGTCCGTATGGCCTGCCCAAATCTTCACTAATGATCCTTCTGCAGGTTCACCTACAGAAACCTTGTTACGACTT
Candida parapsilosis	451	0.00049488	72	optimal	GAATATCTGCAATTCATATTACTTATCGCATTTCGCTGCGTTCTTCATCGATGCGAGAACCAAGAGATCCGTTGTTGAAAGTTTTGACTATTAAATAATCGGTTGACATTAAATAAAATTTGGTTGAGTTTAATCTCTGGCAGGCCCGAAACTTTCACTAATGATCCTTCTGCAGGTTCACCTACAGAAACCTTGTTACGACTT
Penicillium aurantiogriseum	18	1.98E-05	31	optimal	TGAATTTGCAATTCACATTACGTATCGCATTTCGCTGCGTTCTTCATCGATGCCGGAACCAAGAGATCCGTTGTTGAAAGTTTTAAATAATTTATATTTTCACTCAGACTTCAATCTTCAGACAGAGTTCGAGGGTGTCTTCGGCGGGCGCGGGCCCGGGGGCGTGAGCCCCCCCGGCGGCCAGTTAAGGCGGGCCCGCCGAAGCAACAAGGTAAAATAAACACGGGTGGGAGGTTGGACCCAGAGGGCCCTCACTCGGTAATGATCCTTCCGCAGGTTCACCTACGGAAACCTTGTTACGACTT
Malassezia sympodialis	321	0.00035223	1	optimal	GGAATTCTGCAATTCACATTACCTATCGCGTTTCGCTGCGTTCTTCATCGATGGGAGAACCAAGAGATCCGTTGTCGAAAGTTGTGTATAGTTCGTA

					GGCTATACCTACAATTTCACGTACATTCAACCA<br>TACGAGTTTTTTTTGTTAGATGACATTCGTCAT<br>CACAGTGCACAGGGGTTGGTATGGATATAGTG<br>ATGGGCGCCAGAGACGTGTTTGCGTCCGTATG<br>GCAGGCCCGAAACTTTCACTAATGATCCTTCTG<br>CAGGTTCACCTACAGAAACCTTGTTACGACTT
Malassezia sympodialis	300	0.00032919	1	optimal	GGAATTCTGCAATTCACATTACCTATCGCGTTT<br>CGCTGCGTTCTTCATCGATGGGAGAACCAAGA<br>GATCCGTTGTCGAAAGTTGTGTATAGTTCGTA<br>GGCTATACCTACAATTTCACGTACATTCAACCA<br>TACGAGTTTTTTTTGTTCGATGACATTCGTCAT<br>CACAGTGCACAGGGGTTGGTATGGATATAGTG<br>ATTGGCGCCAGAGACGTGTTTGCGTCCGTATG<br>GCAGGCCCGAAACTTTCACTAATGATCCTTCTG<br>CAGGTTCACCTACAGAAACCTTGTTACGACTT
Malassezia sympodialis	452	0.00049598	1	optimal	GGAATTCTGCAATTCACATTACCTATCGCGTTT<br>CGCTGCGTTCTTCATCGATGGGAGAACCAAGA<br>GATCCGTTGTCGAAAGTTGTGTATAGTTAGTA<br>GGCTATACCTACAATTTCACGTACATTCAACCA<br>TACGAGTTTTTTTTGTTCGATGACATTCGTCAT<br>CACAGTGCACAGGGGTTGGTATGGATATAGTG<br>ATGGGCGCCAGAGACGTGTTTGCGTCCGTATG<br>GCAGGCCCGAAACTTTCACTAATGATCCTTCTG<br>CAGGTTCACCTACAGAAACCTTGTTACGACTT
Malassezia sympodialis	277	0.00030395	36	optimal	GAAAATCTGCAATTCATATTACTTATCGCATTT<br>CGCTGCGTTCTTCATCGATGCGAGAACCAAGA<br>GATCCGTTGTTGAAAGTTTTGAAGATTAATTCA<br>ACCATACGAGTTTTTTTTGTTCGATGACATTCG<br>TCATCACAGTGCACAGGGGTTGGTATGGATAT<br>AGTGATGGGCGCCAGAGACGTGTTTGCGTCCG<br>TATGGCAGGCCCGAAACTTTCACTAATGATCCT

					TCTGCAGGTTCACCTACAGAAACCTTGTTACGACTT
Malassezia sympodialis	246	0.00026993	1	optimal	GGAATTCTGCAATTCACATTACCTATCGCGTTTCGCTGCGTTCTTCATCGATGGGAGAACCAAGAGATCCGTTGTCGAAAGTTGTGTATAGTTCGTAGGCTATACCTACAATTTCACGTACATTCAACCATACTAGTTTTTTTTGTTCGATGACATTCGTCATCACAGTGCACAGGGGTTGGTATGGATATAGTGATGGGCGCCAGAGACGTGTTTGCGTCCGTATGGCAGGCCCGAAACTTTCACTAATGATCCTTCTGCAGGTTCACCTACAGAAACCTTGTTACGACTT
Malassezia sympodialis	274	0.00030066	1	optimal	GGAATTCTGCAATTCACATTACCTATCGCGTTTCGCTGCGTTCTTCATCGATGGGAGAACCAAGAGATCCGTTGTCGAAAGTTGTGTATAGTTCGTAGGCTATACCTACAATTTCACGTACATTCAACCATACGAGTTTTTTTTGTTCGATGACATTCGTCATCACAGTGCACAGTGGTTGGTATGGATATAGTGATGGGCGCCAGAGACGTGTTTGCGTCCGTATGGCAGGCCCGAAACTTTCACTAATGATCCTTCTGCAGGTTCACCTACAGAAACCTTGTTACGACTT
Candida metapsilosis	17	1.87E-05	81	optimal	GAATATCTGCAATTCATATTACTTATCGCATTTCGCTGCGTTCTTCATCGATGCGAGAACCAAGAGATCCGTTGTTGAAAGTTTTGACTATTAGTTAATCAAGTTGACAATTAATAAAAAATCCATTTTCAAAATTATTAAATTTCTTTAATGATCCTTCCGCAGGTTCACCTACGGAAACCTTGTTACGACTT
Candida metapsilosis	31	3.40E-05	97	optimal	GAATATCTGCAATTCATATTACTTATCGCATTTCGCTGCGTTCTTCATCGATGCGAGAACCAAGAGATCCGTTGTTGAAAGTTTTGACTATTAGTTAATCAAGTTGACAATTAATAAAAAATTTGGTTGAGTTTAATCTCTGGCAGGCCGTGGGCCCACCAAA

					GCAAAGTTTTTATTAATTGTCAACTTGATTAAC<br>TAATAGTCAAAACTTTCAACCATACGAGTTTTT<br>TTTGTTCGATGACATTCGTCATCACAGTGCACA<br>GGGGTTGGTATGGATATAGTGATGGGCGCCA<br>GAGACGTGTTTGCGTCCGTATGGCAGGCCCGA<br>AACTTTCACTAATGATCCTTCTGCAGGTTCACC<br>TACAGAAACCTTGTTACGACTT

Table S4: Number of sequences retained along DADA2 pipeline for patient samples.

sample	method	input	filtered	denoised1	denoised2	merged	seqtab	nochim
A	optimal	193226	192900	192602	192529	180996	180996	180294
A	standard	193226	12394	12279	12346	11948	11948	11877
B	optimal	284786	282273	281921	282053	279571	279571	279571
B	standard	284786	78625	78472	78524	63776	63776	63776
C	optimal	542213	541630	541384	541201	537585	537585	537505
C	standard	542213	371176	370878	371117	350644	350644	350472
D	optimal	7653	7622	7586	7552	7501	7501	7199
D	standard	7653	3505	3499	3504	3281	3281	3281
E	optimal	1982	1952	1932	1915	1897	1897	1798
E	standard	1982	1352	1341	1347	1328	1328	1328
F	optimal	2527	2494	2478	2437	2429	2429	2366
F	standard	2527	1687	1678	1670	1657	1657	1657

Table S5: Comparison of taxonomic annotation at the Genus and Species level by three versions of the UNITE database and the *assignTaxonomy* function of dada2 by using a seed of 100. UNITE_s: UNITE database including global and 97% singletons; UNITE: UNITE database including singletons set as reference sequences; UNITE+INSD: full UNITE and INSD database. * Top hit is a sequence without Species-level annotation in UNITE. ** Top hit is a sequence with an undefined Species-level annotation in UNITE.

Expected	UNITE_s				UNITE				UNITE+INSD			
	Genus	Bootstrap	Species	Bootstrap	Genus	Bootstrap	Species	Bootstrap	Genus	Bootstrap	Species	Bootstrap
A. fischeri	Aspergillus	99	NA	18	Aspergillus	100	A. fischeri	50	Aspergillus	100	NA	48
A. fumigatus	Aspergillus	100	NA	40	Aspergillus	100	NA	23	Aspergillus	100	A. fumigatus	76
C. albicans	Candida	100	C. albicans	93	Candida	100	C. albicans	99	Candida	100	C. albicans	74
C. glabrata	Nakaseomyces	100	NA*	100	Nakaseomyces	100	NA*	100	Nakaseomyces	95	C. glabrata	95
C. metapsilosis	Candida	99	C. metapsilosis	86	Candida	100	C. metapsilosis	99	Candida	100	C. metapsilosis	100
C. parapsilosis	Candida	100	C. parapsilosis	99	Candida	100	NA	46	Candida	100	C. parapsilosis	100
C. parapsilosis	Candida	100	C. parapsilosis	99	Candida	100	C. parapsilosis	55	Candida	100	C. parapsilosis	100
M. carribica	Meyerozyma	100	M. guilliermondii	97	Meyerozyma	100	M. guilliermondii	92	Meyerozyma	100	M. carpophila	96
M. guillermondii	Meyerozyma	100	M. guilliermondii	99	Meyerozyma	100	M. guilliermondii	100	Meyerozyma	100	NA	46
M. sympodialis	Malassezia	100	M. sympodialis	100	Malassezia	100	M. sympodialis	100	Malassezia	100	M. sympodialis	100
S. cerevisiae	Saccharomyces	100	S. cerevisiae	87	Saccharomyces	100	S. cerevisiae	89	Saccharomyces	100	Saccharomyces species**	100
S. cerevisiae	Saccharomyces	100	S. cerevisiae	95	Saccharomyces	100	S. cerevisiae	92	Saccharomyces	100	Saccharomyces species**	100

Table S6: Tied top hits with lowest e-value returned from a BLAST-based algorithm with the UNITE+INSD database as training set. Occurrences: number of times the same taxonomic annotation was returned. * Taxonomy in the UNITE database has not yet been updated to reflect new nomenclature (Nakaseomyces).

Expected	e-value	Genus	Species	Species-level annotation in UNITE	Occurences
A. fischeri	1.63e-164	Aspergillus	A. fischeri	Yes	16
		Aspergillus	A. lentulus	Yes	2
		Aspergillus	A. arcoverdensis	Yes	2
		Aspergillus	A. oerlinghausenensis	Yes	2
		Aspergillus	Aspergillus sp.	No	8
		Unidentified	Fungi sp.	No	1
A. fumigatus	1.63e-164	Aspergillus	A. fumigatus	Yes	31
		Aspergillus	Aspergillus sp.	No	15
		Unidentified	Fungi sp.	No	3
		Penicillium	Penicillium sp.	No	1
C. albicans	5.14e-139	Candida	C. albicans	Yes	45
		Candida	Candida sp.	No	5
C. glabrata	0	Candida*	C. glabrata	Yes	21
		Nakaseomyces	Nakaseomyces sp.	No	29
C. metapsilosis	1.52e-149	Candida	C. metapsilosis	Yes	45

C. parapsilosis	4.13e-145	Candida	C. parapsilosis	Yes	50
C. parapsilosis	4.13e-145	Candida	C. parapsilosis	Yes	50
M. sympodialis	2.57e-152	Malassezia	M. sympodialis	Yes	44
		Malassezia	Malassezia sp.	No	5
M. caribbica	1.22e-155	Meyerozyma	M. caribbica	Yes	27
		Meyerozyma	M. guilliermondii	Yes	6
		Meyerozyma	M. carbophila	Yes	4
		Meyerozyma	Meyerozyma sp.	No	13
M. guilliermondii	1.22e-155	Meyerozyma	M. guilliermondii	Yes	50
S. cerevisiae	0	Saccharomyces	S. cerevisiae	Yes	10
		Saccharomyces	Saccharomyces sp.	No	38
		Unidentified	Fungi sp.	No	1
		Unidentified	Saccharomycetaceae sp.	No	1
S. cerevisiae	0	Saccharomyces	S. cerevisiae	Yes	3
		Saccharomyces	Saccharomyces sp.	No	45
		Unidentified	Fungi sp.	No	1
		Unidentified	Saccharomcetaceae sp.	No	1

Table S7: Tied top hits with lowest e-value returned from a BLAST-based algorithm with the NT database as training set. Only returned hits with a t least a sequence-based annotation are shown. Occurrences: number of times the same taxonomic annotation was returned.

Expected	e-value	Genus	Species	Occurences
A. fischeri	1.24e-161	Aspergillus	A. fischeri	11
		Aspergillus	A. lentulus	10
		Aspergillus	A. fumigatiaffinis	5
		Aspergillus	A. arcoverdensis	3
		Aspergillus	A. oerlinghausensis	3
		Aspergillus	Aspergillus sp.	2
		Aspergillus	A. novofumigatus	1
A. fumigatus	1.24e-161	Aspergillus	A. fumigatus	49
		Aspergillus	Aspergillus sp.	1
C. albicans	3.87e-136	Candida	C. albicans	45
		Candida	Candida sp.	5
C. metapsilosis	1.15e-146	Candida	C. metapsilosis	8
C. parapsilosis	3.11e-142	Candida	C. parapsilosis	30
C. parapsilosis	3.11e-142	Candida	C. parapsilosis	49
M. sympodialis	1.94e-149	Malassezia	M. sympodialis	19
		Malassezia	M. globosa.	5
M. caribbica	9.19e-153	Meyerozyma	M. caribbica	24
		Meyeroyzma	M. guilliermondii	3

		Meyerozyma	M. carpophila	2
		Meyerozyma	Meyerozyma sp.	1
		Candida	Candida sp.	1
M. guilliermondii	9.19e-153	Meyerozyma	M. guilliermondii	48
		Meyerozyma	M. carpophila	1
		Saccharomyces	Saccharomyces sp.	1
S. cerevisiae	0	Saccharomyces	S. cerevisiae	1024
		Saccharomyces	S. cf cerevisiae/paradoxus	1
S. cerevisiae	0	Saccharomyces	S. cerevisiae	212

Table S8. Tied top hits with lowest e-value returned from a BLAST-based algorithm with the NCBI NT dataset as training set. By the nature of the database, each Species-level annotation has a unique representative sequence available in the NCBI NT database.

Expected	e-value	Genus	Species
A. fischeri	2.60e-166	Aspergillus	A. oerlinghausensis
A. fumigatus	2.60e-166	Aspergillus	A. fumigatus
C. albicans	1.08e-129	Candida	C. africana
C. glabrata	0	Nakaseomyces	C. glabrata
C. metapsilosis	4.09e-139	Candida	C. metapsilosis
C. parapsilosis	2.38e-136	Candida	C. parapsilosis
C. parapsilosis	5.13e-138	Candida	C. parapsilosis
M. sympodialis	5.42e-133	Malassezia	M. sympodialis

M. caribbica	1.92e-157	Meyerozyma	M. caribbica
M. guillliermondii	1.94e-152	Meyerozyma	M. caribbica
S. cerevisiae	0	Saccharomyces	S. arboricola
		Saccharomyces	S. bayanus
		Saccharomyces	S. cariocanus
		Saccharomyces	S. cerevisiae
		Saccharomyces	S. eubayanus
		Saccharomyces	S. kudriavzevii
		Saccharomyces	S. mikatae
		Saccharomyces	S. paradoxus
		Saccharomyces	S. pastorianus
		Saccharomyces	S. uvarum
S. cerevisiae	0	Saccharomyces	S. arboricola
		Saccharomyces	S. bayanus
		Saccharomyces	S. cariocanus
		Saccharomyces	S. cerevisiae
		Saccharomyces	S. eubayanus
		Saccharomyces	S. kudriavzevii
		Saccharomyces	S. mikatae
		Saccharomyces	S. paradoxus
		Saccharomyces	S. pastorianus

		Saccharomyces	S. uvarum

Table S9: Comparison of taxonomic annotation of the Mockrobiota-9 expected sequences at the Genus and Species level by three versions of the UNITE database and the *assignTaxonomy* function of dada2 by using a seed of 100. UNITE_s: UNITE database including global and 97% singletons; UNITE: UNITE database including singletons set as reference sequences; UNITE+INSD: full UNITE and INSD database. * Top hit is a sequence without Species-level annotation in UNITE. ** Top hit is a sequence with an undefined Species-level annotation in UNITE.

Expected	UNITE_s				UNITE				UNITE+INSD			
	Genus	Bootstrap	Species	Bootstrap	Genus	Bootstrap	Species	Bootstrap	Genus	Bootstrap	Species	Bootstra
P. kudriavzevii	Pichia	89	P. kudriavzevii	89	Pichia	99	P. kudriavzevii	99	Pichia	100	P. kudriavzevii	100
P. kudriavzevii	Pichia	88	P. kudriavzevii	88	Pichia	99	P. kudriavzevii	99	Pichia	100	P. kudriavzevii	100
C. jadinii	Cyberlindnera	99	C. jadinii	99	Cyberlindnera	100	C. jadinii	100	Cyberlindnera	100	C. jadinii	100
C. jadinii	Cyberlindnera	100	C. jadinii	100	Cyberlindnera	100	C. jadinii	100	Cyberlindnera	100	C. jadinii	100
C. jadinii	Cyberlindnera	100	C. jadinii	100	Cyberlindnera	100	C. jadinii	100	Cyberlindnera	100	C. jadinii	100
C. jadinii	Cyberlindnera	99	C. jadinii	99	Cyberlindnera	100	C. jadinii	100	Cyberlindnera	100	C. jadinii	100
C. jadinii	Cyberlindnera	100	C. jadinii	100	Cyberlindnera	100	C. jadinii	100	Cyberlindnera	100	C. jadinii	100
H. burtonii	Hypopichia	96	H. burtonii	96	Hypopichia	99	H. burtonii	99	Hypopichia	100	H. burtonii	100
S. fusca	Scopulariopsis	100	S. fusca	83	Scopulariopsis	100	S. fusca	86	Scopulariopsis	100	S. fusca	54
F. domesticum	Fusarium	99	F. domesticum	99	Fusarium	95	F. domesticum	95	Fusarium	100	F. domesticum	100
K. lactis	Kluyveromyces	100	K. lactis	100	Kluyveromyces	100	K. lactis	100	Kluyveromyces	100	K. lactis	100
Z. rouxii	Zygosaccharomyces	100	Z. rouxii	95	Zygosaccharomyces	100	Z. rouxii	100	Zygosaccharomyces	100	NA	57

P. commune	Penicillium	100	NA	23	Penicillium	99	NA	12	Penicillium	99	P. camembertii	57
P. roqueforti	Penicillium	99	P. carneum	98	Penicillium	100	P. roquefortii	88	Penicillium	100	P. carneum	58
D. hansenii	Debaryomyces	100	D. marana	57	Debaryomyces	100	D. hansenii	71	Debaryomyces	100	D. vindobonensis	52
G. candidum	Geotrichum	73	G. candidum	73	Geotrichum	90	G. candidum	90	Geotrichum	90	G. candidum	90
D. catenulata	Diutina	97	D. catenulata	97	Diutina	74	D. catenulata	74	Diutina	100	D. catenulata	100
P. alii	Penicillium	100	P. aurantiogriseum	51	Penicillium	100	P. aurantiogriseum	54	Penicillium	100	P. alii	60

Table S10: Tied top hits with lowest e-value returned from a BLAST-based algorithm with the UNITE+INSD database as training set on the mockrobiota-9 expected sequences. Occurrences: number of times the same taxonomic annotation was returned. * Taxonomy in the UNITE database has not yet been updated to reflect new nomenclature (Nakaseomyces).

Expected	e-value	Genus	Species	Species-level annotation in UNITE	Occurences
P. kudriavzevii	1.61e-118	Pichia	P. kudriavzevii	Yes	42
		Pichia	Pichia sp.	No	7
P. kudriavzevii	1.61e-118	Pichia	P. kudriavzevii	Yes	42
		Pichia	Pichia sp.	No	7
C. jadinii	6.8e-143	Cyberlindnera	C. jadinii	Yes	35
C. jadinii	1.33e-139	Cyberlindnera	C. jadinii	Yes	35
C. jadinii	1.12e-140	Cyberlindnera	C. jadinii	Yes	34
C. jadinii	4.01e-140	Cyberlindnera	C. jadinii	Yes	36
C. jadinii	1.12e-140	Cyberlindnera	C. jadinii	Yes	35
H. burtonii	8.73e-106	Hypopichia	H. burtonii	Yes	49

S. fusca	2.76e-167	Scopulariopsis	S. asperula	Yes	6
		Scopulariopsis	S. fusca	Yes	2
F. domesticum	1.15e-145	Fusarium	F. domesticum	Yes	7
K. lactis	0	Kluyveromyces	K. lactis	Yes	48
		Kluyveromyces	K. dobzhanski	Yes	1
		Kluyveromyces	K. marxianus	Yes	1
Z. rouxii	0	Zygosaccharomyces	Z. rouxii	Yes	9
		Zygosaccharomyces	Zygosaccharomyces sp.	No	41
P. commune	5.71e-159	Penicillium	P. commune	Yes	17
		Penicillium	P. camemberti	Yes	8
		Penicillium	P. cavernicola	Yes	1
		Penicillium	P. palitans	Yes	1
		Penicillium	P. fusoglaucum	Yes	1
		Penicillium	P. echiulatum	Yes	1
		Penicillium	P. paneum	Yes	1
		Penicillium	P. oxalicum	Yes	1
		Penicillium	Penicillium sp.	No	17
		unidentified	Fungi sp.	No	1
		unidentified	Aspergillaceae sp.	No	1
P. roquefortii	7.37e-158	Penicillium	P. roquefortii	Yes	18
		Penicillium	P. carneum	Yes	8
		Penicillium	P. palitans	Yes	1
		Penicillium	Penicillium sp.	No	23

D. hansenii	2.83e-172	Debaryomyces	D. hansenii	Yes	32
		Debaryomyces	D. prospoidis	Yes	2
		Debaryomyces	Debaryomces sp.	No	16
G. candidum	1.37e-98	Geotrichum	G. candidum	Yes	16
D. catenulata	6.54e-102	Diutina	D. catenulate	Yes	49
P. alii	7.39e-158	Penicillium	P. commune	Yes	11
		Penicillium	P. alii	Yes	4
		Penicillium	P. hordei	Yes	2
		Penicillium	P. verrucosum	Yes	1
		Penicillium	P. aurantiogriseum	Yes	1
		Penicillium	Penicillium sp.	No	16
		unidentified	Eurotiales sp.	No	1

Table S11: Tied top hits with lowest e-value returned from a BLAST-based algorithm with the NT database as training set on the mockrobiota-9 expected sequences. Occurrences: number of times the same taxonomic annotation was returned. * Taxonomy in the UNITE database has not yet been updated to reflect new nomenclature (Nakaseomyces).

Expected	e-value	Genus	Species	Species-level annotation in UNITE	Occurences
P. kudriavzevii	1.61e-118	Pichia	P. kudriavzevii	Yes	38
		Pichia	Pichia sp.	No	6
		NA	Uncultured eukaryote	No	1
P. kudriavzevii	1.61e-118	Pichia	P. kudriavzevii	Yes	38
		Pichia	Pichia sp.	No	6
		NA	Uncultured eukaryote	No	1
C. jadinii	5.12e-140	Cyberlindnera	C. jadinii	Yes	1
C. jadinii	1.08e-136	Cyberlindnera	C. jadinii	Yes	1
C. jadinii	8.43e-138	Cyberlindnera	C. jadinii	Yes	4
C. jadinii	3.02e-137	Cyberlindnera	C. jadinii	Yes	1
C. jadinii	8.43e-138	Cyberlindnera	C. jadinii	Yes	1

H. burtonii	6.5e-103	Hypopichia	H. burtonii	Yes	1
S. fusca	2.1e-164	Scopulariopsis	S. asperula	Yes	1
		Scopulariopsis	S. fusca	Yes	1
F. domesticum	8.69e-143	Fusarium	F. domesticum	Yes	2
K. lactis	0	Kluyveromyces	K. lactis	Yes	51
		Kluyveromyces	K. dobzhanski	Yes	1
		Kluyveromyces	K. marxianus	Yes	1
Z. rouxii	0	Zygosaccharomyces	Z. rouxii	Yes	9
			Uncultured fungus	No	10
P. commune	4.34e-156	Penicillium	P. commune	Yes	17
		Penicillium	P. camemberti	Yes	7
		Penicillium	P. fusoglaucum	Yes	2
		Penicillium	P. cavernicola	Yes	1
		Penicillium	P. echinulatum	Yes	1
		Penicillium	P. oxalicum	Yes	1
		Penicillium	P. speluncae	Yes	1
		NA	Fungal sp.	No	3
		Penicillium	Uncultured Penicillium	No	2
P. roquefortii	5.58e-155	Penicillium	P. roquefortii	Yes	13
		Penicillium	P. carneum	Yes	2
		Penicillium	P. palitans	Yes	1
		Penicillium	Penicillium sp.	No	9
		NA	Uncultured fundus	No	1

		Penicillium	Uncultured Penicillium	No	1
D. hansenii	2.15e-169	Debaryomyces	D. hansenii	Yes	40
		Debaryomyces	D. subglobosus	Yes	1
		Debaryomyces	Debaryomces sp.	No	1
		NA	Uncultured fungus	No	1
G. candidum	1.01e-95	Geotrichum	G. candidum	Yes	1
D. catenulata	4.86e-99	Diutina	D. catenulate	Yes	14
P. alii	8e-155	Penicillium	P. alii	Yes	6
		Penicillium	P. hordei	Yes	3
		Penicillium	P. hordei	Yes	2
		Penicillium	P. aurantogriseum	Yes	2
		Penicillium	P. albococremium	Yes	1
		Penicillium	P. verrucosum	Yes	1
		Penicillium	Penciillium sp.	No	3
		NA	Uncultured fungus	No	3

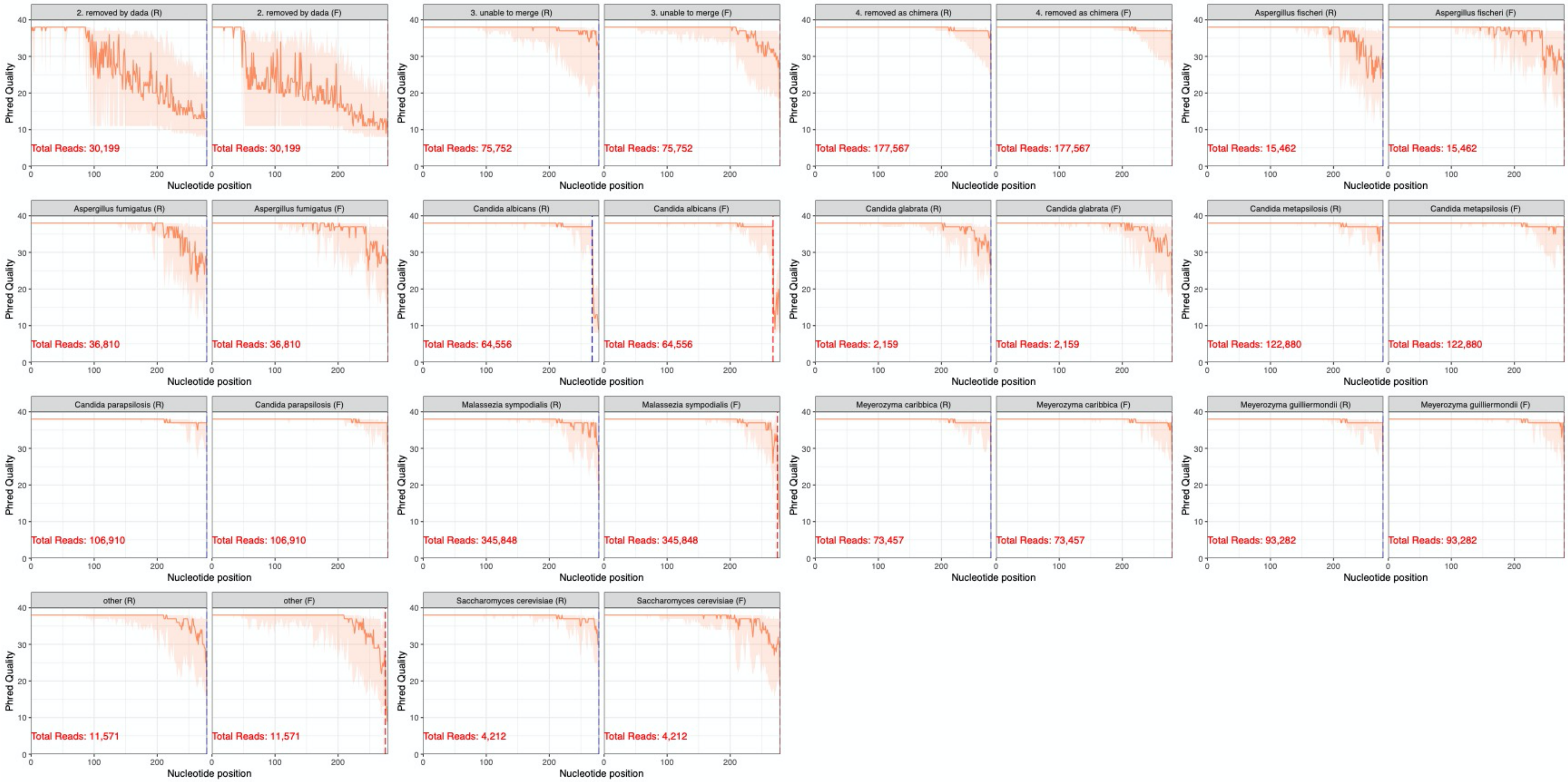

Figure S1. Quality profile of raw reads with different subsequent fates in the DADA2 pipeline using maxEE=Inf, truncQ=0. The orange line represents the median Phred Score at that position, while the shaded area represents the 25th to 75th percentiles. Vertical lines show the median length of reads after primer removal.

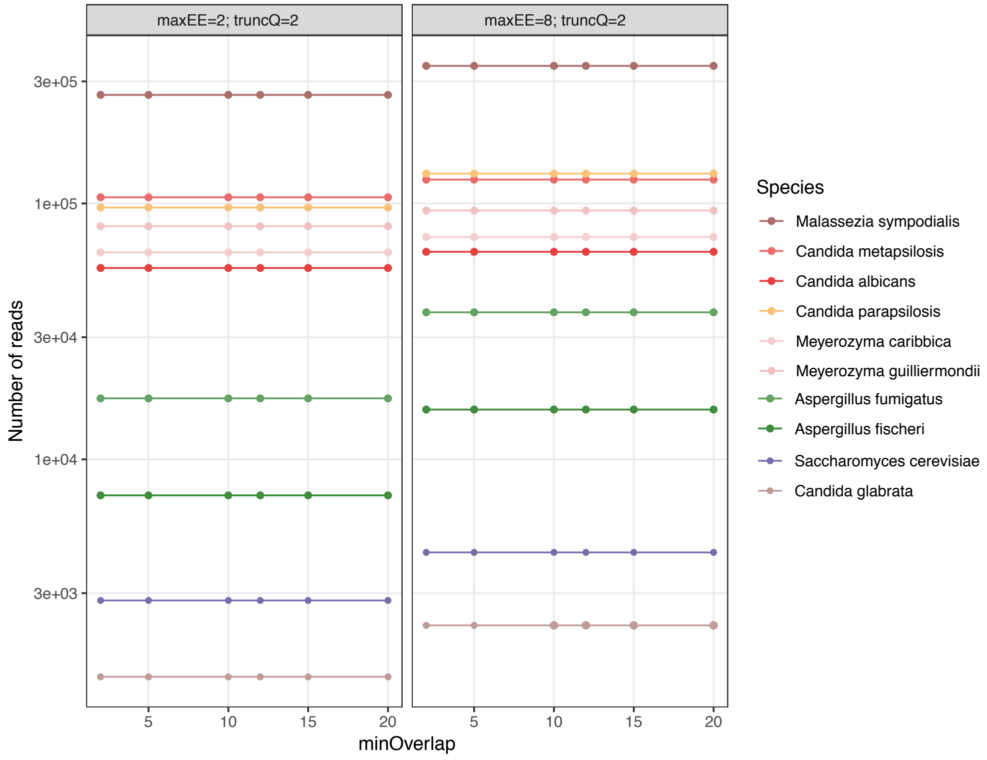

Figure S2. Effect of varying minimal overlap between forward and reverse reads in DADA2 on number of retrieved expected sequences.

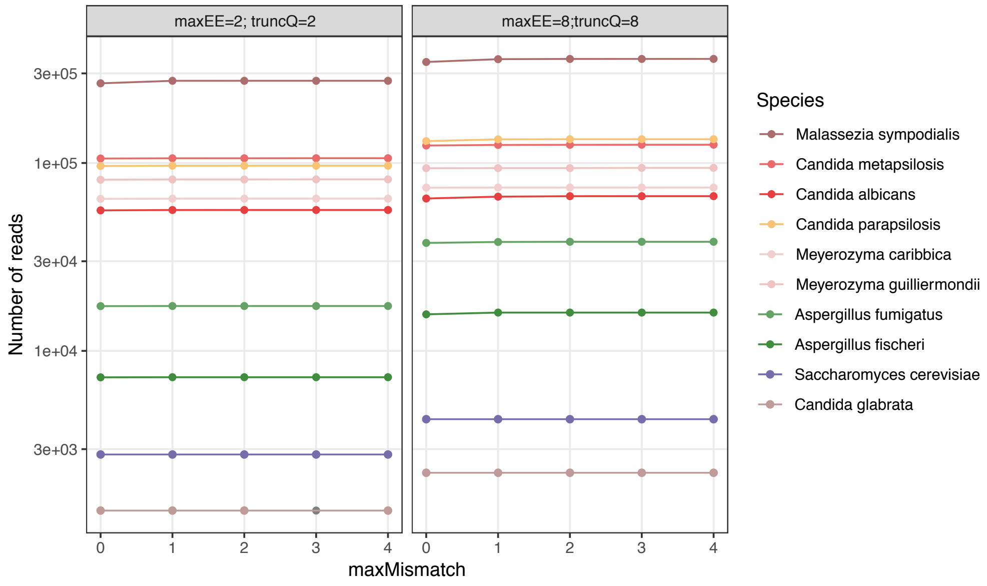

Figure S3. Effect of varying maximum mismatch between forward and reverse reads in DADA2 on number of retrieved expected sequences.
